# Propofol improves survival in a murine model of sepsis via inhibiting Rab5a-mediated intracellular trafficking of TLR4

**DOI:** 10.1186/s12967-024-05107-9

**Published:** 2024-03-28

**Authors:** Bo-Wei Zhou, Wen-Juan Zhang, Fang-Ling Zhang, Xiao Yang, Yu-Qi Ding, Zhi-Wen Yao, Zheng-Zheng Yan, Bing-Cheng Zhao, Xiao-Dong Chen, Cai Li, Ke-Xuan Liu

**Affiliations:** Department of Anesthesiology, Nanfang Hospital, Southern Medical University, Guangzhou, 510515 People’s Republic of China

**Keywords:** Rab5a, TLR4, Sepsis, Propofol

## Abstract

**Background:**

Propofol is a widely used anesthetic and sedative, which has been reported to exert an anti-inflammatory effect. TLR4 plays a critical role in coordinating the immuno-inflammatory response during sepsis. Whether propofol can act as an immunomodulator through regulating TLR4 is still unclear. Given its potential as a sepsis therapy, we investigated the mechanisms underlying the immunomodulatory activity of propofol.

**Methods:**

The effects of propofol on TLR4 and Rab5a (a master regulator involved in intracellular trafficking of immune factors) were investigated in macrophage (from *Rab5a*^−/−^ and WT mice) following treatment with lipopolysaccharide (LPS) or cecal ligation and puncture (CLP) in vitro and in vivo, and peripheral blood monocyte from sepsis patients and healthy volunteers.

**Results:**

We showed that propofol reduced membrane TLR4 expression on macrophages in vitro and in vivo. Rab5a participated in TLR4 intracellular trafficking and both Rab5a expression and the interaction between Rab5a and TLR4 were inhibited by propofol. We also showed Rab5a upregulation in peripheral blood monocytes of septic patients, accompanied by increased TLR4 expression on the cell surface. Propofol downregulated the expression of Rab5a and TLR4 in these cells.

**Conclusions:**

We demonstrated that Rab5a regulates intracellular trafficking of TLR4 and that propofol reduces membrane TLR4 expression on macrophages by targeting Rab5a. Our study not only reveals a novel mechanism for the immunomodulatory effect of propofol but also indicates that Rab5a may be a potential therapeutic target against sepsis.

**Supplementary Information:**

The online version contains supplementary material available at 10.1186/s12967-024-05107-9.

## Background

Sepsis has been defined as life-threatening organ dysfunction caused by a dysregulated host inflammatory immune response to infection, and it remains the leading cause of death in critically ill patients [1]. Many clinical trials have focused on seeking an effective therapeutic target for sepsis, but these have met with very limited success [2, 3]. Therefore, it is imperative to gain a better understanding of the molecular mechanisms involved in the pathogenesis of sepsis and develop novel therapeutic strategies.

Propofol is most widely used in anesthesia induction and maintenance in the operating room and also in sedation for ICU patients including those with sepsis [4]. Previous studies by our group and others have revealed that propofol possesses anti-inflammatory properties in many conditions including sepsis, ischemia/reperfusion injuries of organs, and cardiopulmonary bypass [5–7]. Nevertheless, the precise mechanism that governs these protective effects of propofol is still not well understood.

Sepsis, an inflammatory response triggered by infection, predominantly relies on the innate immune system as its foremost line of defense against foreign bacteria. Macrophages play a crucial role in the clearance of bacteria and responding to the heightened inflammatory state induced by bacterial invasion. Among various immune cells, macrophages play an indispensable role throughout all phases of sepsis due to their ubiquitous presence and comprehensive effects in maintaining immune homeostasis and regulating inflammatory processes. Hence, the scrupulous attention and regulation of macrophage immune function bear immense significance in the realm of sepsis treatment [8, 9]. The toll-like receptor 4 (TLR4) receptor plays an important role in regulating the innate immune response to bacterial infection during sepsis [10, 11]. The activation and upregulation of TLR4 expression on the macrophage cell membrane surface are critical in mediating immuno-inflammatory responses [12]. Most TLR4 molecules are stored in subcellular compartments such as the Golgi apparatus and endosomes, indicating that the expression of cell surface TLR4 is determined by receptor trafficking between the subcellular compartments and the cell membrane [12]. However, the molecular mechanisms of TLR4 intracellular trafficking are unclear. The relationship between propofol and TLR4 has been documented in the literature [13, 14], primarily focusing on the regulatory effects of propofol on the downstream inflammatory pathway associated with TLR4. However, there is a dearth of studies investigating the impact of propofol on TLR4 intracellular trafficking. As propofol is an immunomodulating agent with anti-inflammatory effects, it is reasonable to hypothesize that it may influence cell surface TLR4 expression.

Rab GTPases are a large family of small GTPases that are known as key coordinators of vesicle traffic [15]. Rab5a, one of the Rab GTPases, has been recognized as a master regulator of endocytosis. Rab5a is also involved in the intracellular trafficking of cell surface receptors including TLR4 [16, 17]. However, whether propofol inhibits cell surface TLR4 expression via targeting Rab5a, has not yet been reported.

We hypothesized that Rab5a regulates intracellular trafficking of TLR4 in macrophages and that propofol exerts its anti-inflammatory role by reducing membrane TLR4 expression on macrophages via targeting Rab5a. To investigate these hypotheses, we employed a series of experiments with macrophages following LPS treated in vitro and CLP in vivo. We found that Rab5a is critical in TLR4 intracellular trafficking, and regulates membrane TLR4 expression on macrophages. Propofol exerts its anti-inflammatory role by reducing TLR4 expression on macrophages via inhibiting Rab5a expression and the interaction between Rab5a and TLR4.

## Methods

### Animals

C57/BL mice were obtained from Guangdong Medical Laboratory Animal Center (Guangzhou, China). Rab5a knockout mice were purchased from Cyagen Biosciences (Guangzhou, China). All animal experiments were reviewed and approved by the Ethics Committee of Southern Medical University (approval number NFYY-2019-24).

### BMDM isolation and culture

BMDMs were isolated and cultured as previously described [18]. Briefly, 6–8-week-old male C57BL mice were executed by neck breakage and the tibia and femur were isolated. Bones were kept on ice and rinsed in a sterile dish with DMEM (11971025, Gibco, CA, USA), and bone marrow was then flushed out with DMEM using a 30-gauge needle. Cells were harvested and plated at 10^6^ cell/ml with DMEM containing 10% fetal bovine serum (FBS) (30044333, Gibco, CA, USA), 20% L929 supernatant, 100 U/ml penicillin, and 100 µg/ml streptomycin (15070063, Gibco, CA, USA). The cells were cultured at 37 °C in 5% CO_2_ for 7 days to differentiate into macrophages before further experiments. BMDMs were treated with LPS (1 μg/ml) (L3012, sigma-Aldrich, CA, USA), and propofol (50 μm) (D126608, sigma-Aldrich, CA, USA) were added 30 min before LPS treated according to previous research [19].

### CLP procedure

The CLP-induced mouse sepsis model was performed according to general guidelines [20, 21]. The CLP model involves the perforation of the cecum, which allows for the release of fecal material into the peritoneal cavity, thereby inducing an exaggerated immune response triggered by polymicrobial infection. Male 6**–**8-week-old C57BL mice were anesthetized with isoflurane. For the CLP procedure, in brief, a 2-cm midline laparotomy was performed to expose the cecum. The distal 5 mm of the tip was tightly ligated with 3.0 silk suture and punctured once using an 18-gauge needle. A small amount of fecal material was then squeezed to extrude into the peritoneal cavity. The cecum was returned to the abdominal cavity, and the abdomen and skin were respectively closed using 4.0 silk. Following the surgery, 1 ml of saline was subcutaneously administered to the neck. In the sham group, mice underwent the same procedure, but without the cecal ligation or puncture. In the propofol-treated group, mice were pretreated with 50 mg/kg propofol (dissolved in the fat emulsion) intraperitoneally 30 min before CLP according to previous research [19, 22]. Imipenem (a carbapenem antibiotic with a broader antimicrobial spectrum) was administered subcutaneously at a dose of 0.5 mg/d after CLP [23].

### Tissue histology

After animals were euthanized, segments of the small intestine, lung, liver, and kidney were fixed using 4% paraformaldehyde, embedded in paraffin, used to prepare 5 μm sections, and hematoxylin and eosin (H&E) stained. Two pathologists blinded to study groups then independently evaluated and scored the injury severity of stained sections as in prior studies [21].

### Plasmid transfection

Mouse *Rab5a* (GV141) plasmid was constructed by Genechem (Shanghai, China). BMDMs were transfected with control GV141, or GV141-m*Rab5a* (2.5 μg/10^5^ cells) using jetPEI® reagent (101000053, Polyplus, Strasbourg, France) according to the manufacturer’s protocol. Sequences for plasmids and primers were listed in Additional file 6: Tables S3 and S4. Twenty-four hours after transfection, cells were stimulated with LPS (1 μg/ml) for 24 h and then harvested for membrane TLR4 detection using flow cytometry.

### Immunoprecipitation and immunoblot

Immunoprecipitation and immunoblot were performed as previously described [24]. Cells were lysed in IP lysis buffer (87787, Thermo Scientific, MA, USA) at 4 °C for 30 min. After centrifugation at 12,000×*g* for 20 min, 500 μl supernatant (500 μg protein) was collected and 1 μg TLR4 (ab8376, Abcam, Shanghai, China) antibody was added, followed by incubation overnight at 4 °C. Protein A/G Magnetic beads (20 μl) (88803, Thermo Scientific, MA, USA) were used to capture the protein and antibody complex. The beads were incubated for 4–6 h and washed three times in PBS. Proteins were subjected to SDS-PAGE (6–20% gel) and then transferred to Immobilon-P membranes for Western blotting. For Western blotting, cells were lysed in a radioimmunoprecipitation buffer (P0013B, Beyotime Institute of Biotechnology, Jiangsu, China). The supernatants were obtained by centrifugation at 13,440×*g* for 15 min at 4 °C. The proteins (30 µg/lane) were separated and subsequently transferred onto a polyvinylidene difluoride membrane (IPVH00010, Millipore, MA, USA). The membranes were blocked with 5% non-fat milk at room temperature for 1 h. Following blocking, the membranes were incubated overnight at 4 °C with specific primary antibodies and then incubated with secondary antibodies at room temperature for 1 h. Antibody concentrations used for Western blotting were as follows: rabbit anti-TLR4 (ab22048, Abcam, Shanghai, China) 1:1000; rabbit anti-Na/K ATPase (ab7671, Abcam, shanghai, China) 1:2000; mouse anti-GAPDH (60004-1-Ig, proteintech, Wuhan, China) 1:5000; rabbit anti-p-JNK (4668, Cell Signaling Technology, MA, USA) 1:1000; rabbit anti-p-ERK (4370, Cell Signaling Technology, MA, USA) 1:1000; rabbit anti-RAB5A (11947-1-AP, proteintech, Wuhan, China) 1:1000; The HRP-conjugated anti-mouse secondary antibody (12-349, Sigma-Aldrich, CA, USA) was used at 1:10,000 and the HRP-conjugated anti-rabbit secondary antibody (AP160P, Sigma-Aldrich, CA, USA) was used at 1:5000.

### Immunofluorescence

Cells were fixed and permeabilized with 0.5% TritonX-100, followed by blocking with 1% BSA for 30 min at room temperature. Thereafter, the primary antibodies for TLR4 (ab8376, Abcam, shanghai, China) 1:100, Rab5a (11947-1-AP, Proteintech, Wuhan, China) 1:100, or GM130 (ab52649, Abcam, shanghai, China) 1:200, were added and incubated at 4 °C overnight. After washed 3 times in PBS, cells were incubated with secondary antibody (AlexaFluor 488, 594 goat anti-mouse or anti-rabbit IgG, Invitrogen, 1:200) for 1 h at 37 °C. After being washed 3 times in PBS again and stained with DAPI, immunostaining was observed using an LSM510 (Zeiss) confocal microscope [25].

### Flow cytometric analysis

To detect membrane TLR4 expression and other markers, cells were incubated with fluorescently labeled antibodies against mouse TLR4 (12-9041-80, eBioscience, CA, USA, 1:100) or F4/80 (11-4801-82, eBioscience, CA, USA, 1:500) for 30 min on ice, washed 3 times in PBS, and analyzed in a FACScalibur flow cytometer (BD Bioscience, NJ, USA).

### PCR array

Cytokines and Chemokines PCR Array kit was purchased from Wcgene Biotech (Shanghai China). Each 96-well plate contained containing two housekeeping genes (*Gapdh and Actb*), negative controls, and testing genes. The design of the RT-qPCR array is summarized in Additional file 6: Table S1. The PCR array reaction was carried out in an ABI Q6 Real-Time PCR System (Thermo Fisher Scientific) with the SYBR Green detection protocol (TOYOBO, Osaka, Japan), and analyzed by the standard method of 2^−ΔΔCt^ (Additional file 7).

### Clinical samples

Patients were included if they were at least 18 years of age, and met sepsis criteria within the first 24 h after being admitted to the Intensive Care Unit of Nanfang Hospital. Exclusion criteria were age younger than 18 years, immunosuppressed, treatment with hemodialysis, chemotherapy within 4 weeks, and unable to sign informed consent. A total of 19 septic patients were enrolled. The clinical characteristics, including SOFA score (Sequential Organ Failure Assessment score, for screening sepsis and assessing prognosis), APACHE II score (Acute Physiology and Chronic Health Evaluation, prognostic factors for critically ill patients), causes of sepsis, length of Intensive Care Unit stay, and the 28-day mortality, were recorded in Additional file 6: Table S2. Eleven healthy donors with no medical problems in the medical examination center of Nanfang Hospital were included as controls. Peripheral blood was collected within the first 24 h of Intensive Care Unit admission. Monocytes were prepared as previously described [26]. Human monocyte isolation solution kits (TBD2011H, TBDscience, Tianjin, China) were used for the preliminary isolation of monocytes from peripheral blood before flow cytometry analysis. PE rat anti-human CD14 antibody (301805, Biolegend, CA, USA) was used to mark and sort monocytes. monocytes from healthy volunteers were treated with LPS (1 μg/ml) for the indicated time with or without pretreatment of propofol (50 μM) for 30 min. monocytes from sepsis patients were treated with propofol (50 μM) for 3 h. Expression of TLR4 (APC rat anti-human TLR4 antibody, 312815, Biolegend, CA, USA) on monocyte surface was detected using flow cytometry, and expression of Rab5a mRNA was detected using qPCR. The study protocol was approved by the Ethical Committee of Nanfang Hospital, Southern Medical University (approval number NFEC-202009-k2-01). All individuals gave informed consent to participate.

### Statistical analysis

Results are presented as mean ± SD. Differences between the two groups were analyzed by Student’s t-test. For multi-group comparisons, One-way ANOVA was used followed by Tukey’s post hoc test. Correlation analysis was performed using Pearson correlation. P < 0.05 was considered statistically significant. Graphs and figures were made with Graphpad Prism 5.

## Results

### Propofol inhibits membrane TLR4 expression on macrophages in response to LPS stimulation and CLP

To analyze the expression pattern of cytokines in BMDM upon propofol treatment, we employed cytokines and chemokines PCR array to detect differential expression levels of cytokines in BMDM (the design of the RT-qPCR array is summarized in Additional file 6: Table S1).

**Fig. 1:**
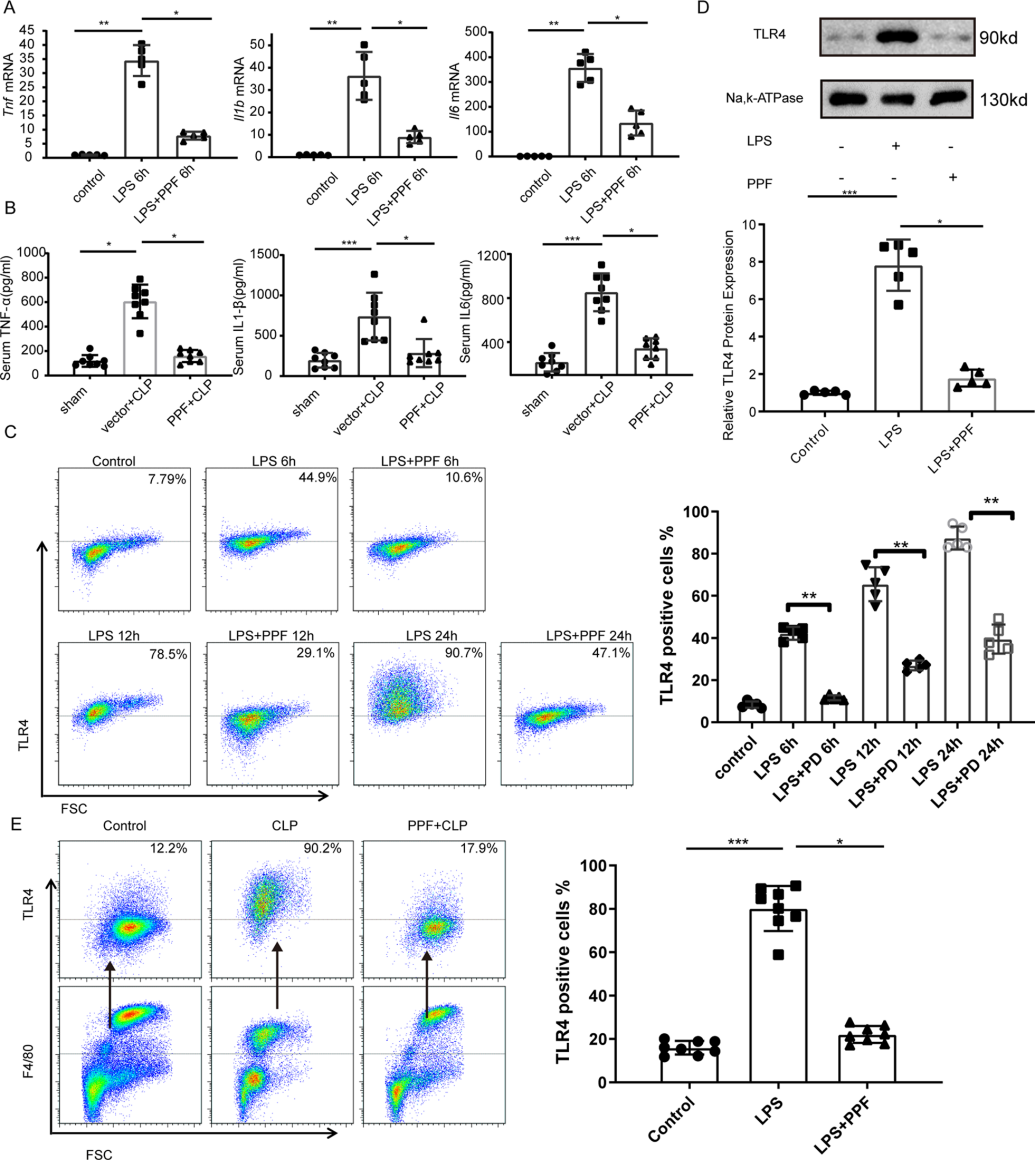
Propofol inhibits membrane TLR4 expression of macrophages after LPS stimulation or CLP. BMDMs were treated with LPS (1 μg/ml) for the indicated time with or without pretreatment of propofol (50 μM) for 30 min. C57BL mice that underwent CLP were pretreated with propofol (50 mg/kg) or equal volume of fat emulsion for 30 min and sacrificed after 12 h. **A** Proinflammatory factor mRNA levels in BMDMs were measured by qPCR. **B** Serum proinflammatory factor levers of CLP mice were assessed by ELISA. **C** Membrane TLR4 expression of BMDMs was analyzed by flow cytometry. **D** Western blot analysis of membrane TLR4 expression of BMDMs. **E** Membrane TLR4 expression of peritoneal macrophages from CLP mice was detected by flow cytometry. Data are expressed as the mean ± SD, n = 5–8, *p < 0.05, **p < 0.01, ***p < 0.001, 1-way ANOVA with Tukey’s post hoc test. *PPF* propofol

As shown in Additional file 1: Fig. S1, the genes with the most significant changes were pro-inflammatory cytokines, including *Il1a*, *il6*, *Il1b,* and *tnf*, which were consistent with previous studies. We further confirmed by qPCR that propofol can effectively inhibit the expression of pro-inflammatory cytokines in BMDMs after LPS treatment, and can reduce the level of pro-inflammatory cytokines in the serum of mice challenged with CLP (Fig. 1A, B). Recent evidence suggests that the amount of TLR4 on the surface of macrophages significantly increases following LPS treatment, reflecting involvement in the inflammatory response [27, 28]. To determine whether propofol reduces plasma membrane TLR4 in macrophages, we measured plasma membrane TLR4 after LPS stimulation using flow cytometry. At 6, 12, and 24 h after LPS stimulation, TLR4 expression on BMDM cell membranes was significantly increased compared to the control group. However, when propofol was added 30 min before LPS stimulation, membrane TLR4 expression on BMDMs decreased significantly at all time points (Fig. 1C). To further confirm these findings, we isolated cytomembrane from BMDMs and then detected the expression of TLR4 by western blotting. As shown in Fig. 1D, propofol significantly inhibited the cell surface expression of TLR4 in response to LPS. Interestingly, propofol does not affect the total expression of TLR4 as shown in Additional file 2: Fig. S2.

To explore the influence of propofol on macrophage plasma membrane TLR4 in vivo, we performed standardized CLP on male mice, the most reliable model in sepsis research. Propofol (50 mg/kg) was injected intraperitoneally 30 min before CLP. Six hours after surgery, peritoneal macrophages were collected and marked with the cell surface marker F4/80. Similar to the vitro results, CLP induced an increase in the amount of TLR4 on the peritoneal macrophage surface, and propofol eliminated the TLR4 response otherwise induced by CLP (Fig. 1E). Moreover, propofol significantly reduced organ injury in CLP mice, indicated by hematoxylin and eosin [H&E] staining and serum markers (Additional file 3: Fig. S3A, B). Propofol also increased the survival of CLP mice (Additional file 3: Fig. S3C). Taken together these results indicate that propofol reduces the amount of cell surface TLR4 induced by immune challenge.

### Knockout of Rab5a attenuates membrane TLR4 expression on macrophages and inhibits the activation of macrophages in response to LPS and CLP

**Fig. 2:**
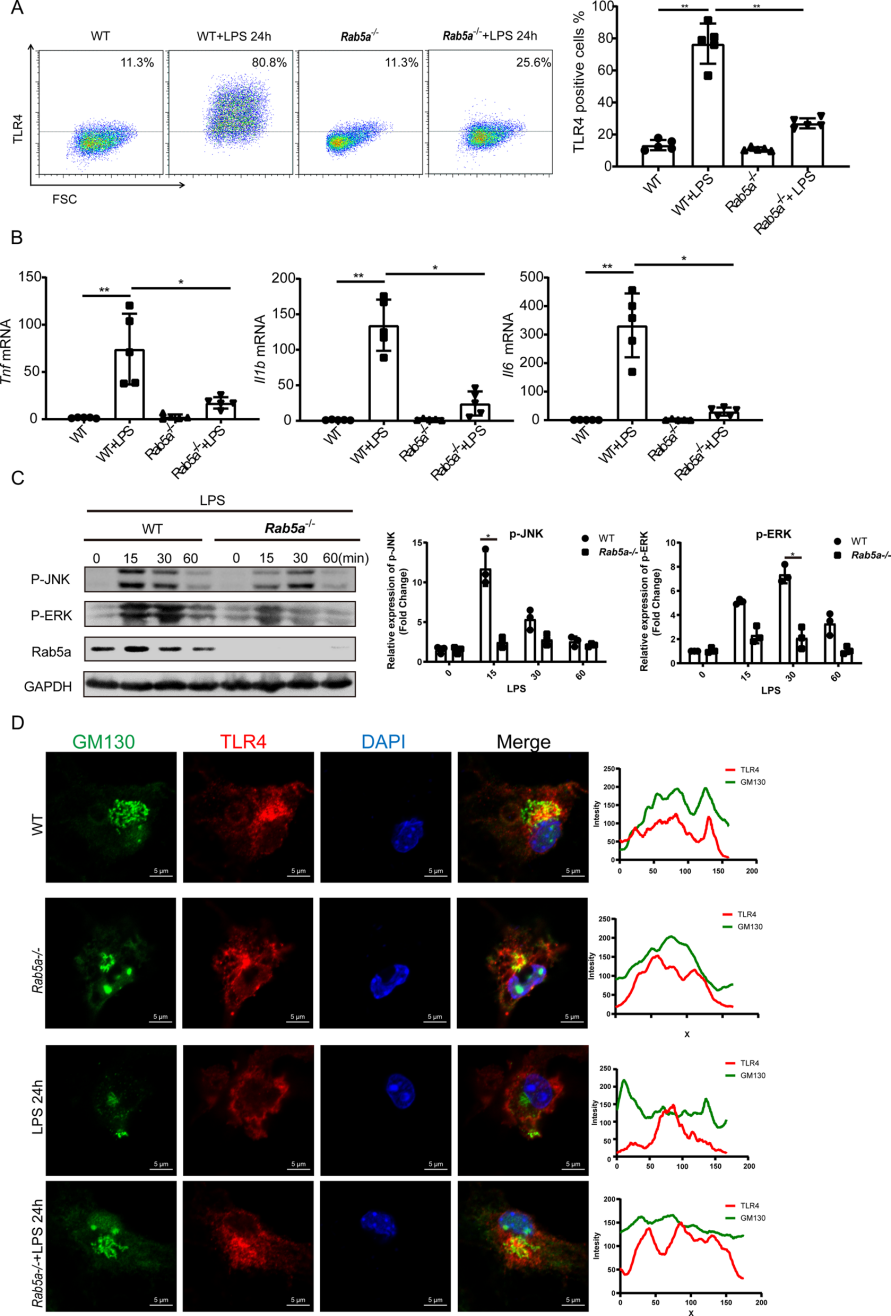
Knockout of *Rab5a* attenuates membrane TLR4 expression on macrophages and inhibits the activation of macrophages. BMDMs from wild-type and *Rab5a*^−/−^ C57BL mice were treated with LPS (1 μg/ml) for the indicated time. **A** Membrane TLR4 expression of BMDMs was analyzed by flow cytometry. **B** Proinflammatory factor mRNA levels in BMDMs were measured by qPCR. **C** Western blotting of MAPK levels in BMDMs. **D** Representative image of TLR4 (red) and GM130 (green) immunofluorescence staining in BMDMs, scale bars: 5 μm. Data are expressed as the mean ± SD, n = 5, *p < 0.05, **p < 0.01, ***p < 0.001, 1-way ANOVA with Tukey’s post hoc test

**Fig. 3:**
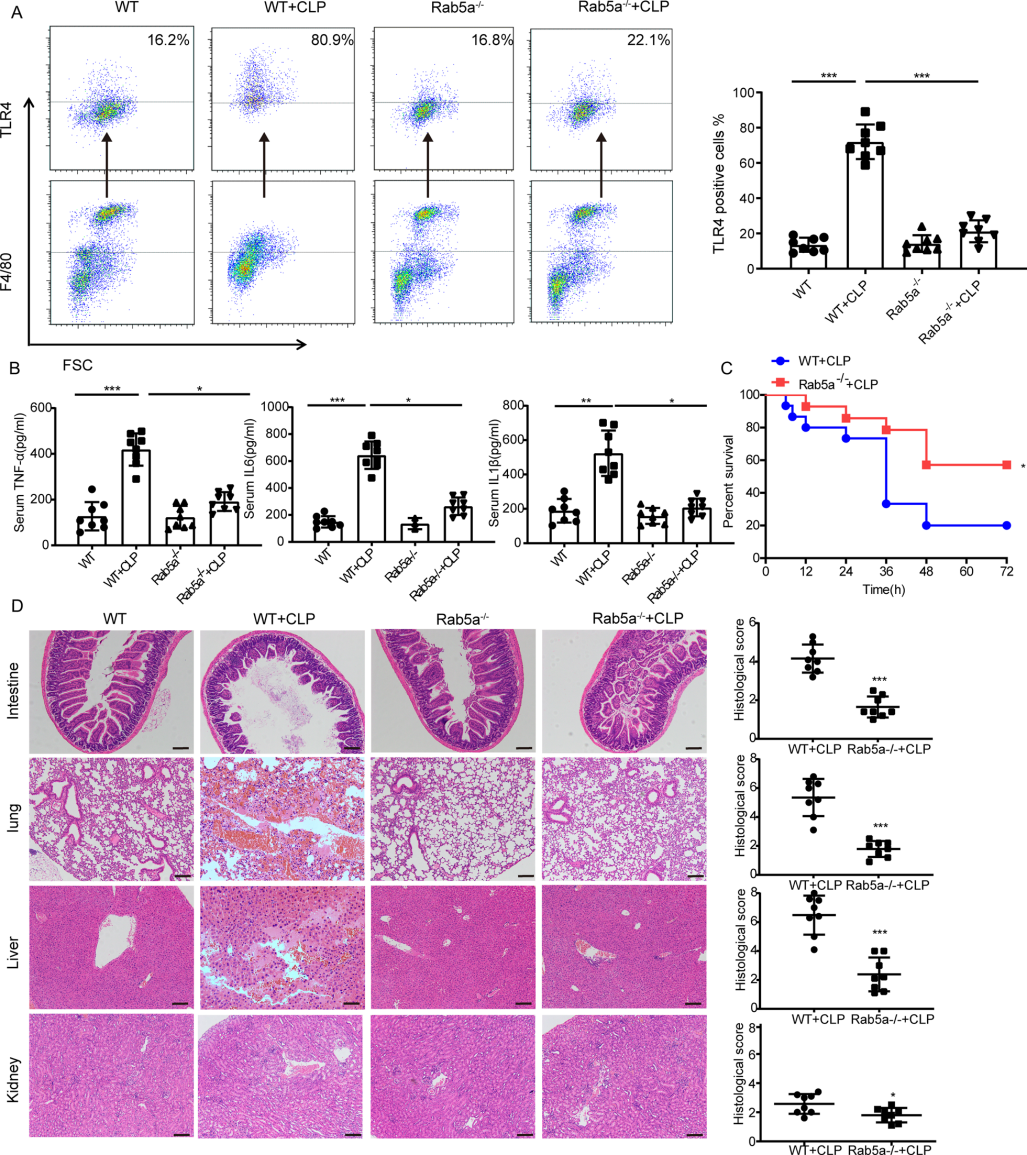
Knockout of *Rab5a* attenuates membrane TLR4 expression on macrophages. Wild type and *Rab5a*^−/−^ mice that underwent CLP were sacrificed after 12 h. **A** Membrane TLR4 expression of peritoneal macrophages from CLP mice was detected by flow cytometry. **B** Serum proinflammatory factor levers of CLP mice were assessed by ELISA. **C** The survival curve of Wild type and *Rab5a*^−/−^ mice underwent CLP. Kaplan Meier analysis was used to evaluate the survival rate of CLP mice (n = 15). **D** H&E staining and histological score of lung, liver, kidney, and intestinal in wild type and *RAB5a*^*−*/−^ mice, Scale bars: 50 μm. Data are expressed as the mean ± SD, n = 8, *p < 0.05, **p < 0.01, ***p < 0.001, unpaired t test or 1-way ANOVA with Tukey’s post hoc test

Since propofol reduces the amount of cell surface TLR4 without affecting the total expression of TLR4, we hypothesize that propofol may regulate the intracellular transport of TLR4. It is well established that Rab5a is a key regulator of endocytosis and transportation of plasma membrane compartments [29, 30]. Therefore, we asked whether Rab5a participates in the transport of TLR4 and/or regulates the expression of TLR4 on the cell membrane. Flow cytometry results revealed that TLR4 expression on the cell surface of BMDMs from *Rab5a*^−/−^ mice was significantly lower than that in the WT group after LPS stimulation (Fig. 2A). Furthermore, knockout of Rab5a inhibited the expression of proinflammatory cytokines and activation of the JNK and ERK (belong to the MAPK pathway and play a key role in inflammatory response during sepsis) in BMDMs upon LPS treated (Fig. 2B, C). In vivo, we also found a decreased release of proinflammatory cytokines in the serum of *Rab5a*^−/−^ mice compared to WT mice subjected to CLP (Fig. 3B). We further identified an interaction between TLR4 and Rab5a by co-immunoprecipitation and confocal immune-fluorescence microscopy. LPS promoted interaction and co-localization between TLR4 and Rab5a (Fig. 3B–D). To confirm the role of Rab5a in TLR4 intracellular trafficking, we labeled the Golgi apparatus in BMDMs with GM130 antibody. We found that TLR4 was partially localized in the Golgi before LPS stimulation. LPS weakened the co-localization of TLR4 and Golgi, indicating a translocation of TLR4 from Golgi to the cell membrane, and this response was attenuated by *Rab5a* knockout (Fig. 2D).

To further investigate the role of Rab5a in septic mice, we performed CLP on *Rab5a*^*−/−*^ mice. Peritoneal macrophages from *Rab5a*^*−/−*^ CLP mice showed reduced cell surface TLR4 expression compared to the WT group (Fig. 3A). The expression of serum cytokines in *Rab5a*^*−/−*^ CLP mice was also significantly lower than in WT CLP mice (Fig. 3B). Further, the survival of *Rab5a*^*−/−*^ CLP mice was significantly increased compared to that of the WT group, and histopathological sections suggested that the damage to lung, liver, kidney, and intestinal tissue in *Rab5a*^*−/−*^ mice with CLP surgery was significantly alleviated compared to WT mice (Fig. 3C, D). Taken together, these data indicate that Rab5a is essential for the intracellular trafficking of TLR4 from Golgi to the cell membrane, and for the upregulation of cell surface TLR4 upon LPS treatment. Knockdown of Rab5a can attenuate the inflammatory response and organ damage in septic mice, suggesting it is a potential target for treating sepsis.

### Propofol inhibits Rab5a expression and the interaction between Rab5a and TLR4 in macrophages in response to LPS and CLP

**Fig. 4:**
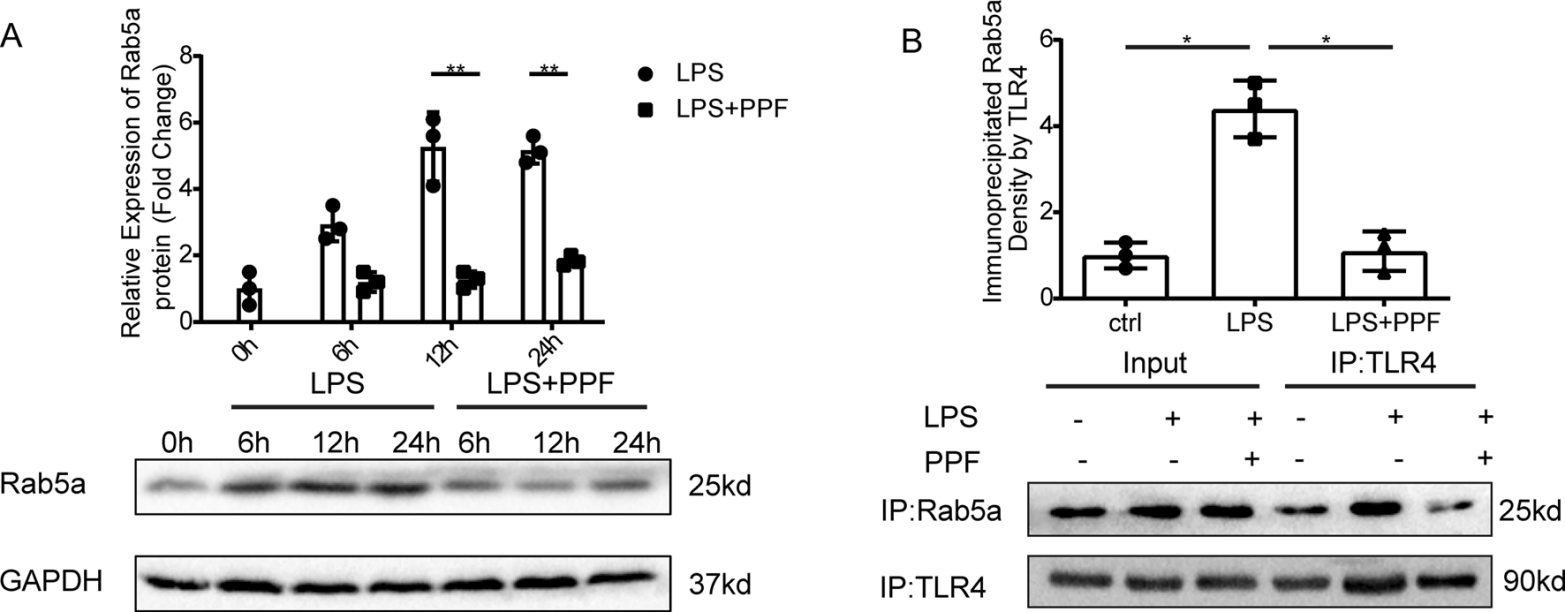
Propofol inhibits Rab5a expression and the interaction between Rab5a and TLR4 in macrophages. BMDMs were treated with LPS (1 μg/ml) for the indicated time with or without pretreatment of propofol (50 μM) for 30 min. **A** Western blotting of Rab5a in BMDMs. **B** Interaction of TLR4 withRab5a determined by Co-IP analyses in BMDMs. Immunoprecipitation (IP) and immunoblotting (IB) were performed with anti-TLR4 and anti-Rab5a antibodies. Data are expressed as the mean ± SD, n = 3, *p < 0.05, **p < 0.01, ***p < 0.001, 1-way ANOVA with Tukey’s post hoc test

Rab5a has previously been shown to play a significant role in membrane receptor trafficking [31]. We hypothesized that Rab5a may bind to TLR4 and help it to traffic between membrane and cytoplasm during LPS treatment and that this may be suppressed by propofol. We first evaluated the expression of Rab5a in BMDMs treated with LPS, with or without propofol pretreatment. Western blotting analysis showed that LPS increased the expression of Rab5a, and this effect was suppressed by propofol pretreatment (Fig. 4A) and we found that propofol did not affect Rab7 and Rab10 (Additional file 2: Fig. S2C). Co-immunoprecipitation revealed that propofol pretreatment weakened the interaction between TLR4 and Rab5a (Fig. 4B). Collectively, these data reveal that propofol inhibits both Rab5a expression and the interaction between Rab5a and TLR4.

### Propofol reduces membrane TLR4 expression on macrophages upon LPS treatment through downregulating Rab5a

To gain further insight into whether propofol inhibits cell surface TLR4 expression by LPS-treated macrophages through regulating Rab5a, we analyzed the amount of cell surface TLR4 in *Rab5a*-overexpressing BMDMs in response to LPS in the presence or absence of propofol pretreatment. As shown in Additional file 4: Fig. S4A, B, the amount of cell surface TLR4 was increased by Rab5a upregulation and abolished by propofol pretreatment in BMDM treated with LPS compared to an empty-vector-transfected group (Additional file 5: Fig. S5). Furthermore, propofol could not effectively reduce the expression of pro-inflammatory factors in Rab5a-overexpressing BMDMs treated with LPS compared to the control group (Additional file 4: Fig. S4C).

In summary, these results reveal that propofol attenuates the expression of cell surface TLR4 by downregulating Rab5a.

### Propofol inhibits the expression of Rab5a and TLR4 on LPS-treated peripheral blood monocytes from healthy volunteers as well as septic patients

**Fig. 5:**
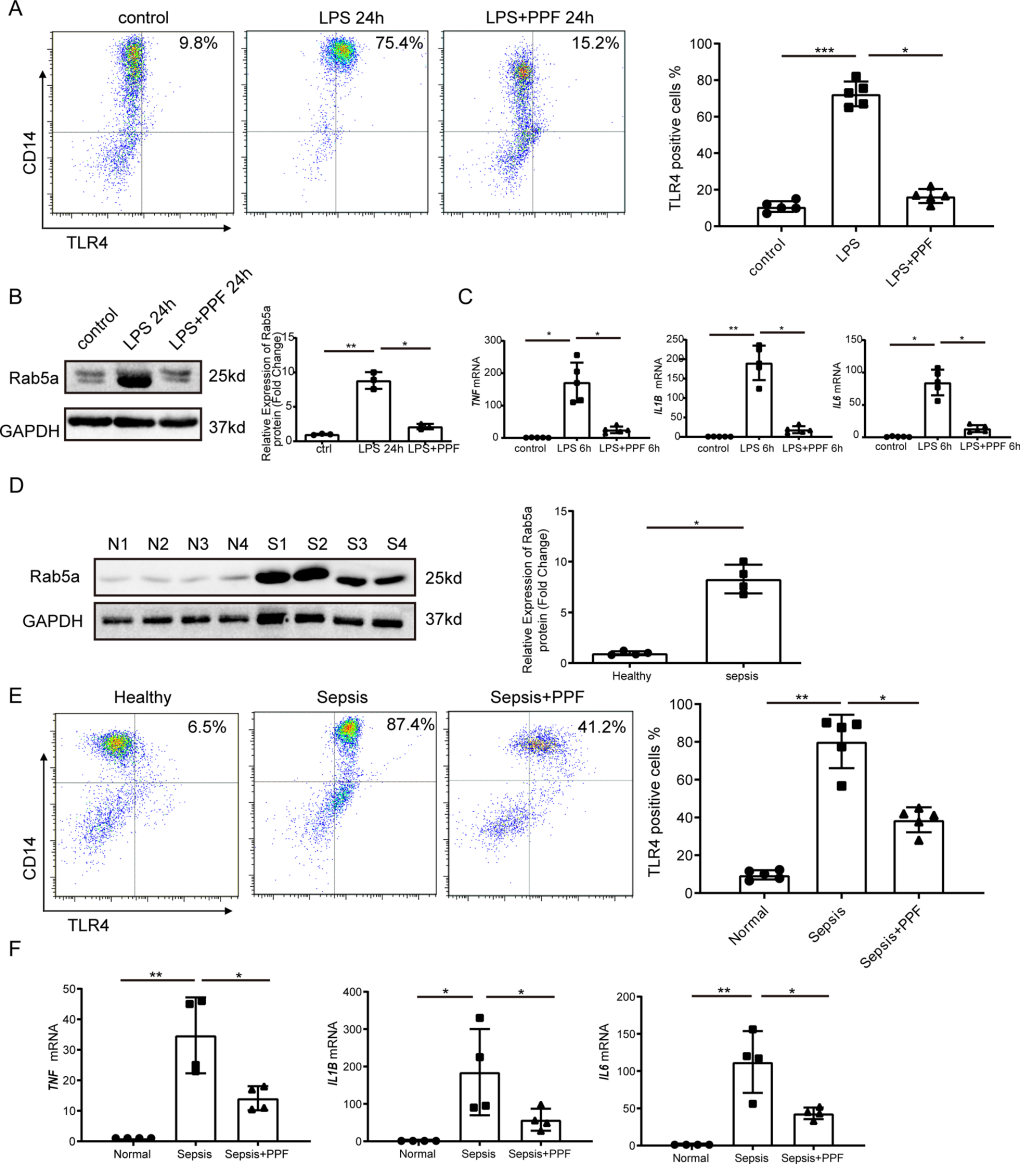
Propofol inhibits the expression of Rab5a and TLR4 on LPS-treated peripheral blood monocytes. peripheral blood monocytes from healthy volunteers were treated with LPS treated with LPS (1 μg/ml) for the indicated time with or without pretreatment of propofol (50 μM) for 30 min. **A** Membrane TLR4 expression of peripheral blood monocytes from healthy volunteers was analyzed by flow cytometry. CD14 antibody was used to mark monocytes. **B** Western blotting of Rab5a in peripheral blood monocytes from healthy volunteers. **C** Proinflammatory factor mRNA levels in peripheral blood monocytes from healthy volunteers were measured by qPCR. **D** Western blotting of Rab5a in peripheral blood monocytes from healthy volunteers and sepsis patients. N1 to N4 represent healthy volunteers and S1 to S4 represent sepsis patients. **E** Membrane TLR4 expression of peripheral blood monocytes from healthy volunteers and sepsis patients were measured by flow cytometry. Peripheral blood monocytes from sepsis patients were treated with propofol (50 μM) for 3 h. **F** Proinflammatory factor mRNA levels in peripheral blood monocytes from healthy volunteers and sepsis patients were measured by qPCR. Peripheral blood monocytes from sepsis patients were treated with propofol (50 μM) for 3 h. Data are expressed as the mean ± SD, n = 3–5, *p < 0.05, **p < 0.01,***p < 0.001, 1-way ANOVA with Tukey’s post hoc test. *PPF* propofol

**Fig. 6:**
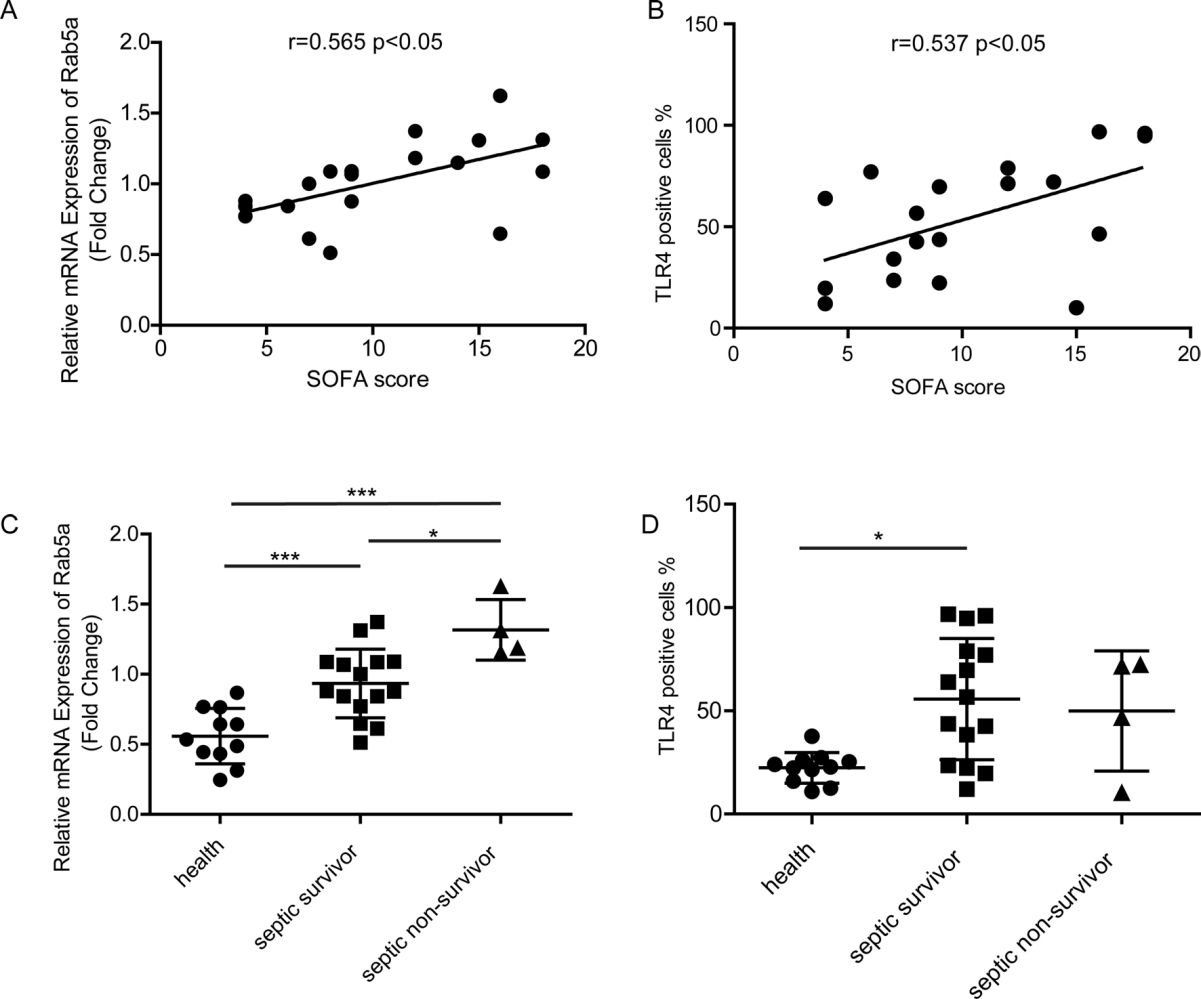
Rab5a expression in peripheral blood monocytes from sepsis patients and its clinical relevance. **A** The correlation between Rab5a mRNA level in peripheral blood monocytes from sepsis patients and SOFA scores. The data were analyzed using the Pearson correlation test (r = 0.565, P < 0.05, n = 19). **B** The correlation between membrane TLR4 in peripheral blood monocytes from sepsis patients and SOFA scores. The data were analyzed using the Pearson correlation test (r = 0.537, P < 0.05, n = 19). **C** Rab5a mRNA level in peripheral blood monocytes from health control, septic survivor, and septic non-survivor (n = 11, 15, 4). **D** Membrane TLR4 in peripheral blood monocytes from health control, septic survivor, and septic non-survivor (n = 11, 15, 4). Data are expressed as the mean ± SD, *p < 0.05, **p < 0.01, ***p < 0.001, 1-way ANOVA with Tukey’s post hoc test

In order to validate the results detailed above with in vitro and in vivo experiments, and to exclude differences between mice and humans, we extracted peripheral blood monocytes from healthy volunteers and verified the effects of propofol on Rab5a and TLR4 following LPS stimulation. We found that the expression of Rab5a and TLR4 on the surface of human peripheral blood monocytes increased after LPS stimulation and propofol pretreatment inhibited the expression of Rab5a and cell surface TLR4 (Fig. 5A, B). In addition, lower levels of mRNA expression of proinflammatory cytokines were also observed in the propofol pretreatment group (Fig. 5C). Further, we extracted peripheral blood mononuclear cells in sepsis patients and found a significant increase in both Rab5a and cell surface TLR4 expression compared with Health volunteers. Propofol was able to inhibit the expression of Rab5a and cell surface TLR4, and the release of inflammatory factors (Fig. 5D–F). Moreover, Rab5a and membrane TLR4 expressions were positively associated with the Sequential Organ Failure Assessment score of sepsis patients (Fig. 6A, B), and more importantly, the septic non-survivors had significantly increased Rab5a mRNA levels when compared to the septic survivors (Fig. 6C). The above findings showed that the expression levels of Rab5a and TLR4 are elevated in peripheral blood mononuclear cells of patients with sepsis, and the mechanisms by which propofol exerts its anti-inflammatory effects include inhibiting the expression of Rab5a and TLR4, which is in accordance with the results of our in vitro and in vivo experiments.

## Discussion

In this study, we found that possibly as a result of its involvement in TLR4 intracellular trafficking, Rab5a is critical for macrophage activation and TLR4-mediated inflammatory reaction.

Another important finding of this study was that propofol exerts its anti-inflammatory role by reducing membrane TLR4 expression on macrophages via inhibiting Rab5a expression and the interaction between Rab5a and TLR4. Consistently, we also showed that both Rab5a, and cell surface TLR4 expression, were upregulated in LPS-treated peripheral blood monocytes from healthy volunteers, and in peripheral blood monocytes from septic patients, and that expression of both factors was downregulated by propofol. The correlation between the expression of both factors and the SOFA score of sepsis patients and the high expression of Rab5a in septic non-survivors peripheral blood monocytes further enhanced the clinical relevance of our study. In this study, we demonstrated for the first time, that propofol, acting as an immunomodulating agent, exerts its anti-inflammatory role in sepsis by targeting Rab5a and thereby downregulating membrane TLR4 expression by macrophages.

TLR4 is a key signaling molecule in initiating the immune response to exogenous bacteria and endogenous damage-associated molecular patterns during endotoxemia or sepsis. It can be activated by LPS, a major component of the cell walls of gram-negative bacteria, heat shock proteins released by necrotic cells, heparin sulfate, polysaccharide degraded from hyaluronate, and other factors [32]. Numerous studies have suggested that TLR4 is a “double-edged sword” during sepsis [32, 33]. On the one hand, it can mediate the inflammatory reaction to remove exogenous microorganisms and damage cells in the body. On the other hand, excessive inflammation can cause damage to normal tissue and may lead to multiple organ failure. Therefore, as a crucial regulation molecule of endotoxemia or sepsis, TLR4 expression on the cell membrane surface is tightly regulated [34, 35]. Here, we showed that propofol prevents excessive activation of inflammatory responses by reducing TLR4 expression on the surface of macrophages exposed to exogenous bacteria. Therefore, propofol may be a useful immunomodulating agent to return an aberrant immune response to homeostasis in sepsis.

It has been demonstrated that the expression of cell-surface TLR4 is determined by both endocytosis of cell-surface TLR4 into endosomes and receptor trafficking from the Golgi apparatus to the cell membrane [36]. However, the molecular events involved in the membrane trafficking of TLR4 are still not well understood. Many studies have shown that LPS not only causes endocytic degradation of TLR4 but also promotes intracellular TLR4 transport to the cell membrane [37, 38]. Rab5a has been recognized as a master regulator of endocytosis and is involved in the intracellular trafficking of cell surface receptors [16]. Of interest, we showed that knockout of the Rab5a gene not only reduces endocytosis of TLR4 but also abolishes the replenishment of TLR4 from Golgi to the cell membrane. This indicates that Rab5a is a key molecule in the membrane trafficking of TLR4 for its role in bridging TLR4 endocytosis and replenishment. Rab5a has been documented to participate in the trafficking of various cell surface receptors such as TLR2, C5ar1, and EGFR, among others, via endosomes and other intracellular vesicles [39–41]. However, limited studies have explored the interplay between receptors regulated by Rab5a. Is there a competitive inhibition relationship between different receptors in binding with Rab5a, for example, TLR2 and TLR4. Alternatively, it can be achieved through synergistic effects, such as the existing literature suggesting a correlation between EGFR receptor trafficking and TLR4 receptor activation [42]. Moreover, distinct receptor trafficking may be governed by the same signaling pathway. Existing literature suggests that both TLR2 and C5ar1 receptors are regulated by the Rab5a-PI3K signaling axis [39, 40]. CD14 and integrin β3 are also involved in the intracellular trafficking of TLR4, especially in endocytosis. The requirement of Rab5a involvement in the transport of those receptors remains to be further investigated [43]. The utilization of immuno-electron microscopy can enable observation of TLR4 trafficking between intracellular vesicles and organelles while further investigating how Rab5a facilitates its trafficking mechanism. Furthermore, employing Co-IP combined mass spectrometry might help identify additional signaling molecules involved in Rab5a and TLR4 trafficking.

Sepsis is defined as a life-threatening organ dysfunction caused by a dysregulated host response to infection [44]. In sepsis, the immune response that is initiated by an invading pathogen is refractory to a return to homeostasis, thus culminating in a pathological syndrome that is characterized by sustained excessive inflammation and immune suppression [45]. Immunomodulation has been considered an important avenue of treatment for sepsis, but anti-inflammatory agents against inflammatory factors have been shown to fail to improve patient outcomes [46]. New therapeutic targets and individualized treatment options are urgently needed. Our study revealed that propofol inhibits the interaction between TLR4 and Rab5a and abolishes Rab5a-mediated TLR4 membrane trafficking, indicating that propofol may enact its anti-inflammatory properties by mediating TLR4 expression via targeting of Rab5a. Moreover, higher expression of Rab5a and surface TLR4 were found in peripheral blood monocytes of patients with sepsis than in the normal control group, and both were correlated with the SOFA score of sepsis patients, with a higher expression of Rab5a found in septic non-survivors. Given the importance of these factors to the immune response, therapeutic methods focusing on exploring the possible mechanisms involved in TLR4 trafficking, the regulation of Rab5a expression, and the amount of TLR4 on macrophage surfaces, may be effective strategies for treating sepsis.

However, our clinical data suggest that TLR4 expression is not associated with patient survival, and studies of eritoran (a TLR-4 antagonist) have failed in clinical trials [47], which may raise questions about the clinical application of our findings. As TLR4 not only mediates inflammatory responses leading to organ damage but also plays an important role in maintaining the normal function of the immune system, direct blocking of TLR4 may not benefit all patients. Based on the failure of the eritoran clinical trial, some scholars proposed new strategies for enrolling only patients who are likely to respond to the drug based on appropriate biochemical screening [48]. Rab5a is elevated and is associated with survival in sepsis patients. Given that Rab5a is critical in TLR4 trafficking, high expression of Rab5a may indicate abnormal transport and overactivation of TLR4. Therefore, Rab5a may be a good marker for screening patients sensitive to TLR4 inhibitors.

Limitations of this study include our inability to explore the exact mechanisms of propofol’s effect on Rab5a. Propofol was initially defined as a gamma-aminobutyric acid (GABA) receptor antagonist. Recent studies have demonstrated that immune cells, including macrophages, also express GABA receptors [49]. The function of GABA in the immune system is at an early stage of study, but inhibitory effects have been observed in autoimmune diseases. The question of whether propofol regulates Rab5a through GABA receptors, is of interest for future research.

## Conclusions

**Fig. 7:**
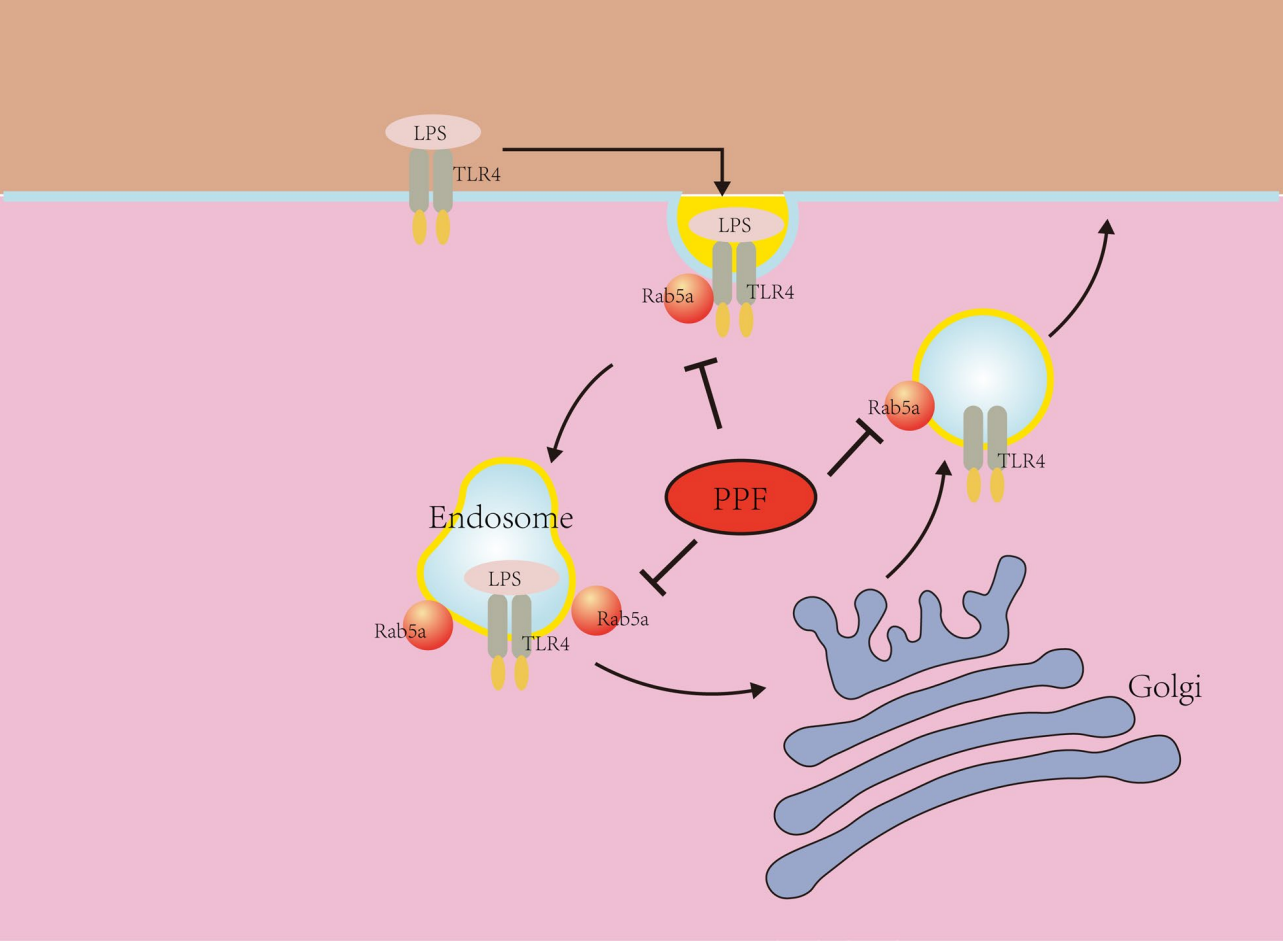
Schematic representation of the potential mechanism of propofol in regulating membrane TLR4 expression on macrophages. Rab5a participates in intracellular trafficking of TLR4 between Golgi and cell membrane in macrophages upon LPS treated and propofol reduces membrane TLR4 expression on macrophages by inhibiting Rab5a expression and the interaction between Rab5a and TLR4

In conclusion, we demonstrated that Rab5a regulates intracellular trafficking of TLR4 and propofol reduces membrane TLR4 expression on macrophages by targeting Rab5a (Fig. 7). Our study sheds light on the mechanism underlying the anti-inflammatory effect of propofol from the aspects of receptor transport and immune regulation. This informs the potential clinical application of propofol to septic patients and indicates that Rab5a may be a potential therapeutic target against sepsis.

### Supplementary Information

**Additional file 1: Figure S1.** Cytokines and Chemokines PCR Array in BMDMs. (A) Heatmap showed differentially expressed genes in BMDMs by RT-RT-qPCR array. (B) The volcano plot analyzed the significantly up-(red) and down-regulated (green) based on fold-change (FC ≥ |2| and p < 0.05). The statistical model used for the volcano map data was negative binomial models, and the test method was generalized linear models, Benjamini and Hochberg for adjustments were made for multiple comparisons. (C) Differential expression analysis of DEGs.

**Additional file 2: Figure S2.** Propofol does not affect the total expression of TLR4 in BMDMs. BMDMs were treated with LPS (1 μg/ml) for the indicated time with or without pretreatment of propofol (50 μM) for 30 min. (A) Western blotting of TLR4 in BMDMS. (B) The comparison of total TLR4 expression in BMDMs. Data are expressed as the mean ± SD, n = 3, NS: No Significant Difference, 1-way ANOVA with Tukey’s post hoc test. PPF: propofol.

**Additional file 3: Figure S3.** Propofol reduces organ damage and improves survival in sepsis mice. C57BL mice that underwent CLP were pretreated with propofol (50 mg/kg) or an equal volume of fat emulsion for 30 min. (A) H&E staining and histological score of lung, liver, kidney, and intestinal of CLP mice. The mice were sacrificed 24 h after CLP (n = 8). (Scale bars: 50 μm). (B) Serum ALT, AST, and Scr levers of CLP mice. The mice were sacrificed 24 h after CLP (n = 8). (C) The survival curves of CLP mice. Kaplan Meier analysis was used to evaluate the survival rate of CLP mice (n = 20). Data are expressed as the mean ± SD, *p < 0.05, **p < 0.01, ***p < 0.001, unpaired t-test or 1-way ANOVA with Tukey’s post hoc test. PPF: propofol.

**Additional file 4: Figure S4.** Propofol reduces membrane TLR4 expression on macrophages upon LPS treatment through downregulating Rab5a. BMDMs were transfected with GV141-m*Rab5a* or empty vector and stimulated with LPS (1 μg/ml) for 24 h with or without pretreatment of propofol (50 μM) for 30 min. (A) The overexpression efficiency of *Rab5a* was confirmed by western blotting. (B) membrane TLR4 expression of BMDMs was analyzed by flow cytometry. Data are expressed as the mean ± SD, n = 3–5, *p < 0.05, **p < 0.01,***p < 0.001, 1-way ANOVA with Tukey’s post hoc test. PPF: propofol, EV: empty vector, OV: Overexpression.

**Additional file 5: Figure S5.** Flow cytometric gating strategy.

**Additional file 6: Table S1.** Cytokines and Chemokines PCR Array. **Table S2.** Characteristics of sepsis patients and health control. **Table S3.** Sequences of primers used for qRT-PCR. **Table S4.** Sequences for plasmids.

**Additional file 7.** Raw data for RT-PCR array.

## Data Availability

All datasets used and/or analyzed supporting the conclusions are available from the corresponding author upon reasonable request.

## Abbreviations

CLP: Cecal ligation and puncture
LPS: Lipopolysaccharide
PBS: Phosphate-buffered saline
GABA: Gamma-aminobutyric acid
TLR4: The toll-like receptor 4
BMDM: Bone marrow-derived macrophages
PBMC: Peripheral blood monocyte
MAPK: Mitogen-activated protein kinases
H&E: Hematoxylin and eosin
PCR: Polymerase chain reaction
