# Supplementary figures and images for: Propofol improves survival in a murine model of sepsis via inhibiting Rab5a-mediated intracellular trafficking of TLR4
Source: J Transl Med. 2024 Mar 28;22:316. doi: 10.1186/s12967-024-05107-9 (PMC10976826; doi:10.1186/s12967-024-05107-9)

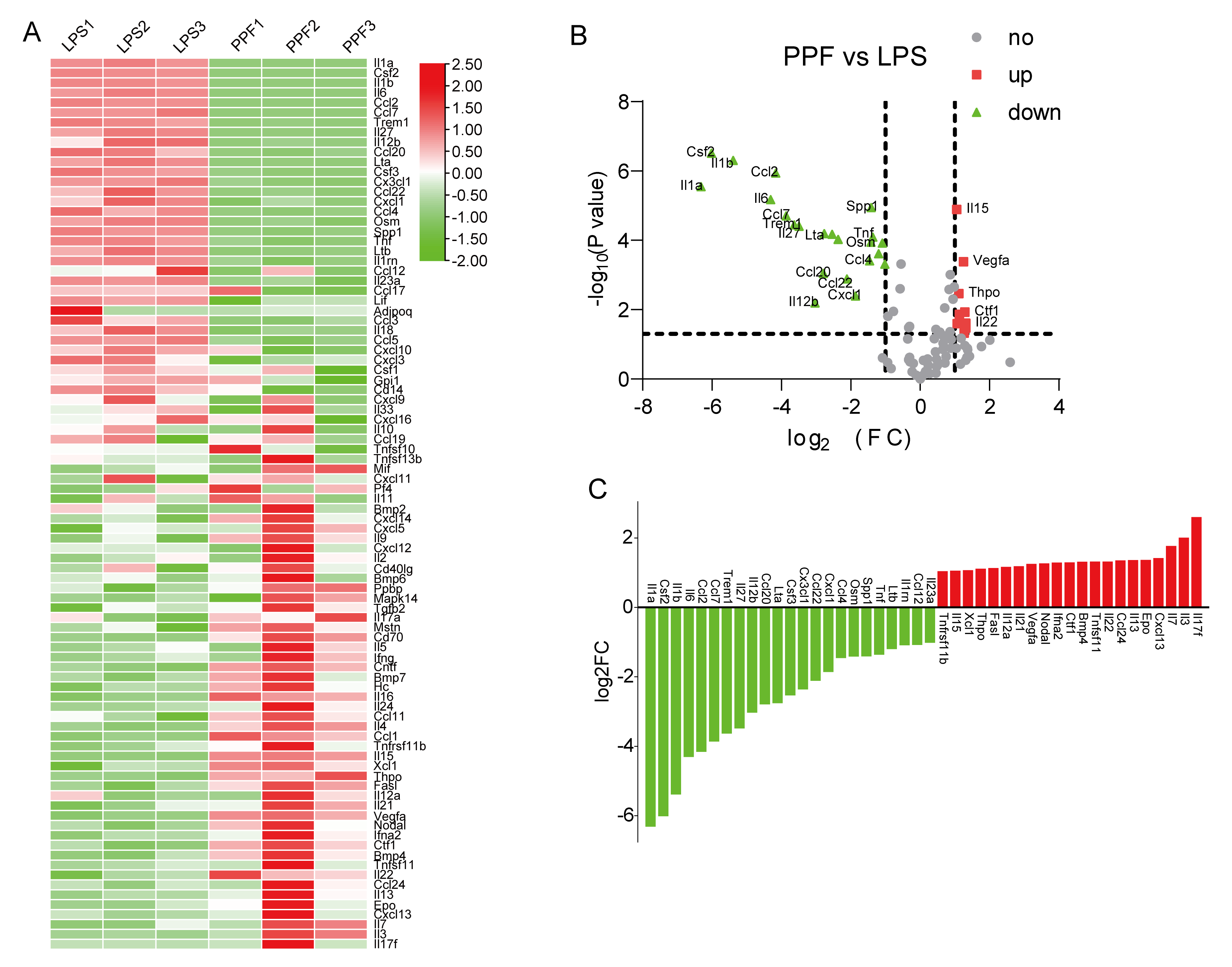

Supplement: Supplementary file 1 — Additional file 1: Figure S1. Cytokines and Chemokines PCR Array in BMDMs. (A) Heatmap showed differentially expressed genes in BMDMs by RT-RT-qPCR array. (B) The volcano plot analyzed the significantly up-(red) and down-regulated (green) based on fold-change (FC ≥ |2| and p < 0.05). The statistical model used for the volcano map data was negative binomial models, and the test method was generalized linear models, Benjamini and Hochberg for adjustments were made for multiple comparisons. (C) Differential expression analysis of DEGs. [file 12967_2024_5107_MOESM1_ESM.tif]

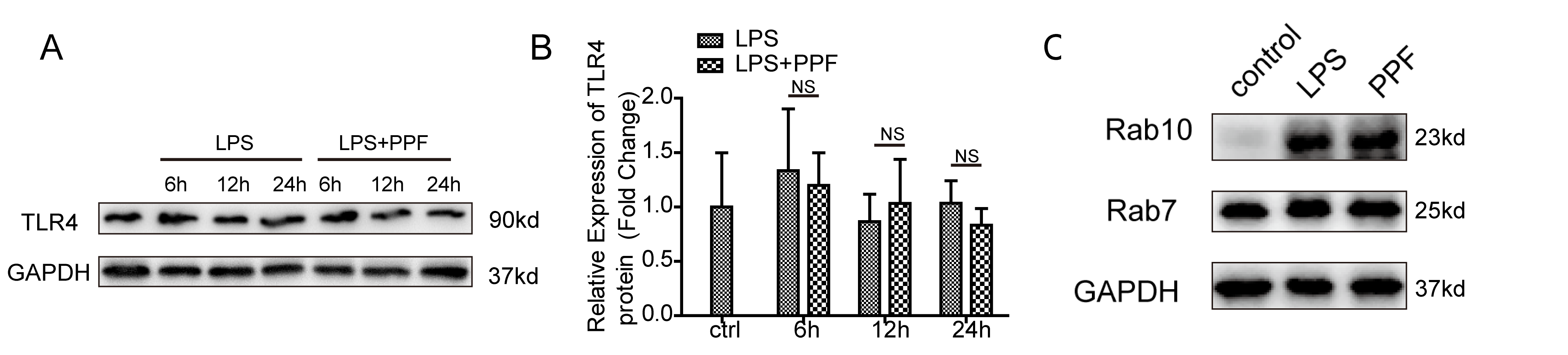

Supplement: Supplementary file 2 — Additional file 2: Figure S2. Propofol does not affect the total expression of TLR4 in BMDMs. BMDMs were treated with LPS (1 μg/ml) for the indicated time with or without pretreatment of propofol (50 μM) for 30 min. (A) Western blotting of TLR4 in BMDMS. (B) The comparison of total TLR4 expression in BMDMs. Data are expressed as the mean ± SD, n = 3, NS: No Significant Difference, 1-way ANOVA with Tukey’s post hoc test. PPF: propofol. [file 12967_2024_5107_MOESM2_ESM.tif]

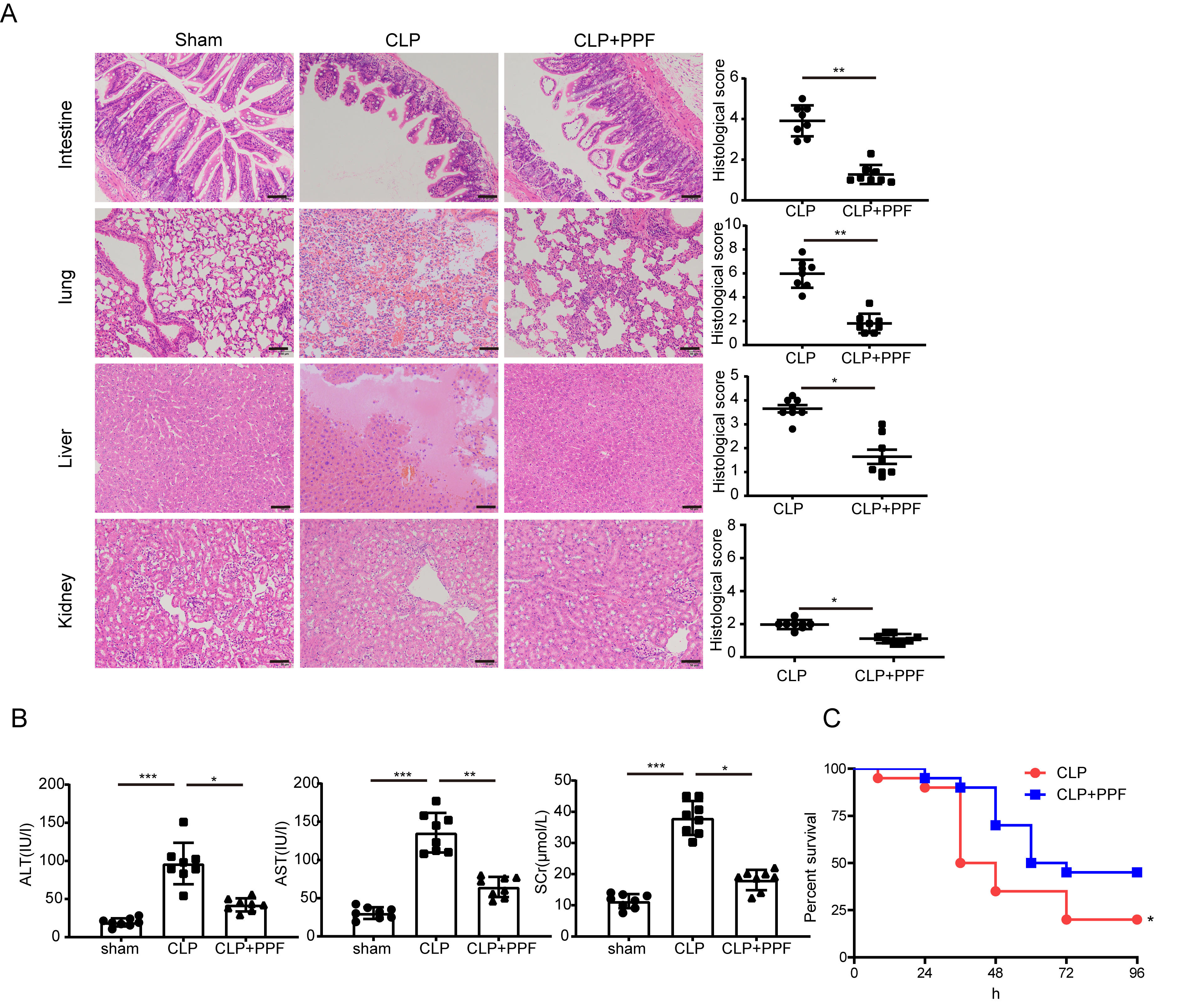

Supplement: Supplementary file 3 — Additional file 3: Figure S3. Propofol reduces organ damage and improves survival in sepsis mice. C57BL mice that underwent CLP were pretreated with propofol (50 mg/kg) or an equal volume of fat emulsion for 30 min. (A) H&E staining and histological score of lung, liver, kidney, and intestinal of CLP mice. The mice were sacrificed 24 h after CLP (n = 8). (Scale bars: 50 μm). (B) Serum ALT, AST, and Scr levers of CLP mice. The mice were sacrificed 24 h after CLP (n = 8). (C) The survival curves of CLP mice. Kaplan Meier analysis was used to evaluate the survival rate of CLP mice (n = 20). Data are expressed as the mean ± SD, *p < 0.05, **p < 0.01, ***p < 0.001, unpaired t-test or 1-way ANOVA with Tukey’s post hoc test. PPF: propofol. [file 12967_2024_5107_MOESM3_ESM.tif]

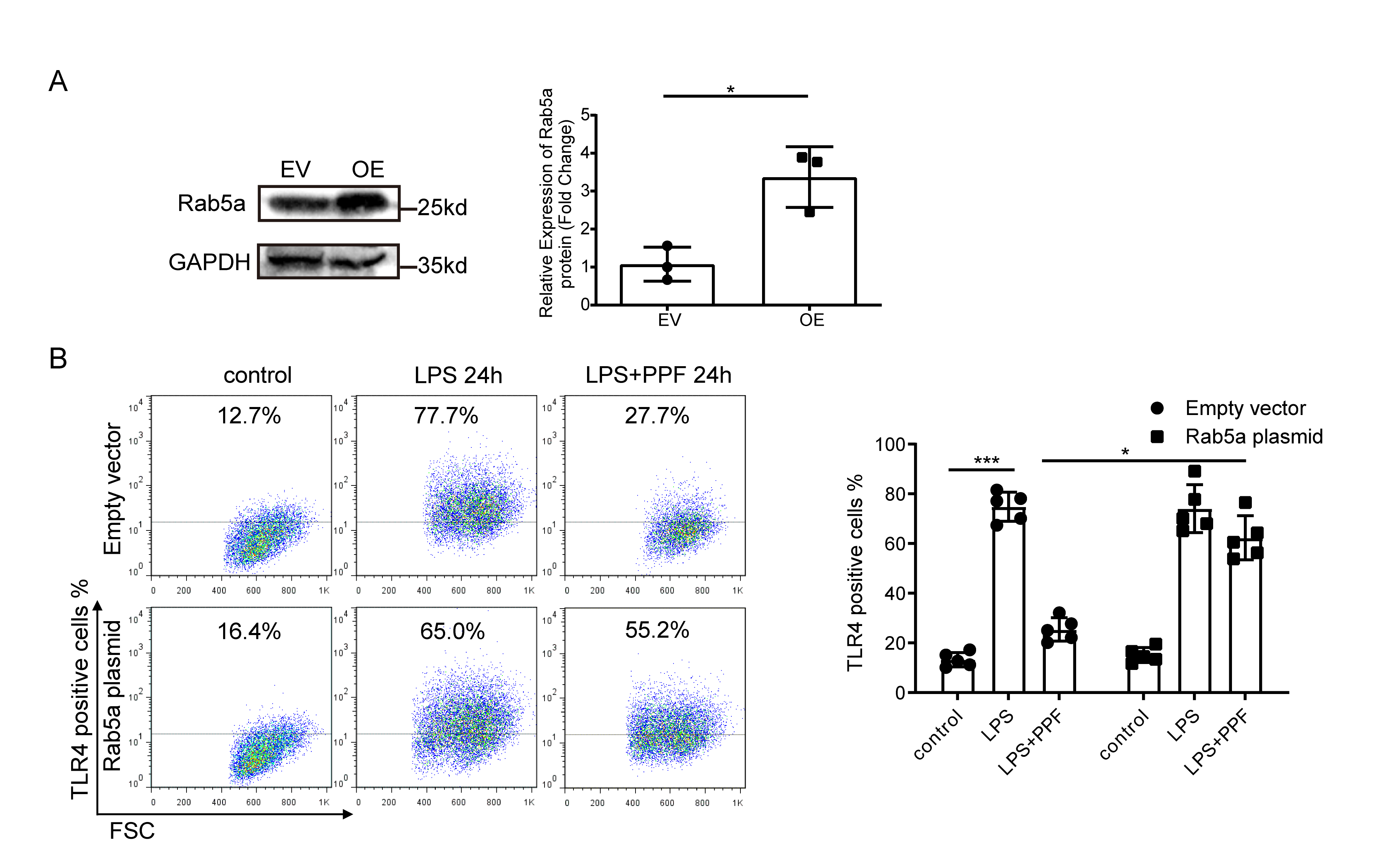

Supplement: Supplementary file 4 — Additional file 4: Figure S4. Propofol reduces membrane TLR4 expression on macrophages upon LPS treatment through downregulating Rab5a. BMDMs were transfected with GV141-mRab5a or empty vector and stimulated with LPS (1 μg/ml) for 24 h with or without pretreatment of propofol (50 μM) for 30 min. (A) The overexpression efficiency of Rab5a was confirmed by western blotting. (B) membrane TLR4 expression of BMDMs was analyzed by flow cytometry. Data are expressed as the mean ± SD, n = 3–5, *p < 0.05, **p < 0.01,***p < 0.001, 1-way ANOVA with Tukey’s post hoc test. PPF: propofol, EV: empty vector, OV: Overexpression. [file 12967_2024_5107_MOESM4_ESM.tif]

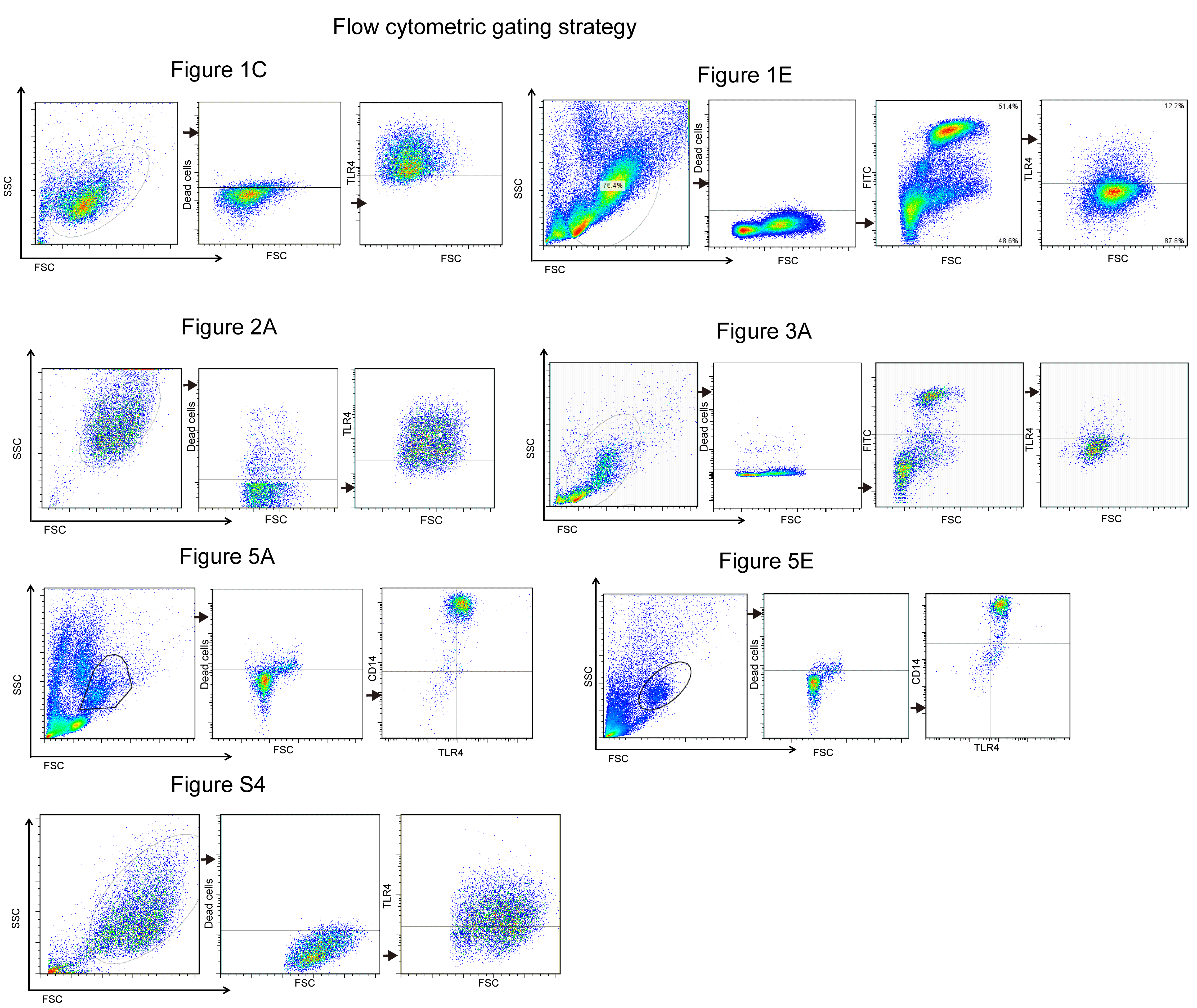

Supplement: Supplementary file 5 — Additional file 5: Figure S5. Flow cytometric gating strategy. [file 12967_2024_5107_MOESM5_ESM.tif]
