# Supplementary material for: Propofol improves survival in a murine model of sepsis via inhibiting Rab5a-mediated intracellular trafficking of TLR4
Source: J Transl Med. 2024 Mar 28;22:316. doi: 10.1186/s12967-024-05107-9 (PMC10976826; doi:10.1186/s12967-024-05107-9)
Supplement: Supplementary file 6 — Additional file 6: Table S1. Cytokines and Chemokines PCR Array. Table S2. Characteristics of sepsis patients and health control. Table S3. Sequences of primers used for qRT-PCR. Table S4. Sequences for plasmids. [file 12967_2024_5107_MOESM6_ESM.docx]

**Table S1. Cytokines & Chemokines PCR Array**

|  | Gene name |
| --- | --- |
| chemokines | *Ccl1, Ccl11, Ccl12, Ccl17, Ccl19, Ccl2, Ccl20 ,Ccl22, Ccl24, Ccl3, Ccl4, Ccl5, Ccl7, Cx3cl1, Cxcl1, Cxcl10, Cxcl11, Cxcl12, Cxcl13, Cxcl16, Cxcl3, Cxcl5, Cxcl9, Pf4, Ppbp, Xcl1* |
| interleukin | *Il10, Il11, Il12a, Il12b, Il13, Il15, Il16, Il17a, Il17f, Il18, Il1a, Il1b, Il1rn, Il2,Il21, Il22, Il23a, Il24, Il27, Il3, Il4, Il5, Il6, Il7, Il9* |
| interferon | *Ifna2,Ifng* |
| Member of the TNF receptor superfamily | *Cd40lg, Cd70, Fasl, Lta, Ltb, Tnf, Tnfrsf11b, Tnfsf10, Tnfsf11, Tnfsf13b* |
| Other cytokines | *Adipoq,Ctf1,Hc,Mif,Spp1,Tgfb2* |

**Table S2. Characteristics of sepsis patients and health control**

| Characteristics | Septic Survivors  (n=15) | Septic Non-survivors （n=4） | Health  (n=11) | P value |
| --- | --- | --- | --- | --- |
| Age (yr) | 57.73±15.57 | 63.25±13.35 | 52.27±14.77 | 1 |
| Sex, male (%) | 11 (73.33%) | 3 (75%) | 5 (45.45%) | 0.91 |
| APACHE II score | 20.40±5.49 | 31.25±6.09 |  | <0.01 |
| SOFA score | 9.27±4.73 | 14.25±1.71 |  | 0.06 |
| Sepsis due to |  |  |  |  |
| Peritonitis | 2 (13.33%) | 2 (50%) |  |  |
| Pneumonia | 5 (33.33%) | 2 (50%) |  |  |
| Urinary tract infections | 1 (6.67%) | 0 |  |  |
| Skin and soft tissue infections | 4 (26.67%) | 0 |  |  |
| Other | 3 (20%) | 0 |  |  |
| Length of ICU stay | 11.4±12.71 | 15.25±6.13 |  | 0.57 |

**Table S3.** **Sequences of primers used for qRT-PCR**

| Gene name | Sequence (5’-3’) |
| --- | --- |
| TLR4(forward) | AACGCTTCACGAATTTGCGT |
| TLR4(reverse) | GCTTCGGCAGCACATATACTAA |
| Rab5a (forward) | TACAACTGCCCCTAGCGGT |
| Rab5a (reverse) | TGAGGAAAACGAGTGCAAACAT |
| IL1-b(forward) | GAAACAACCGACGAGAGTCTG |
| IL1-b (reverse) | TGTGTGGTCTTGCATTCATGG |
| TNF-α (forward) | CCCTCACACTCAGATCATCTTCT |
| TNF-α (reverse) | GCTACGACGTGGGCTACAG |
| IL-6 (forward) | TAGTCCTTCCTACCCCAATTTCC |
| IL-6 (reverse) | TTGGTCCTTAGCCACTCCTTC |
| 18S (forward) | ACACGGACAGGATTGACAGA |
| 18S (reverse) | GGACATCTAAGGGCATCACA |

**Table S4. Sequences for plasmids**

| **Plasmids** | **Sequence (5’-3’)** |
| --- | --- |
| GV141  gacggatcgggagatctcccgatcccctatggtgcactctcagtacaatctgctctgatgccgcatagttaagccagtatctgctccctgcttgtgtgttggaggtcgctgagtagtgcgcgagcaaaatttaagctacaacaaggcaaggcttgaccgacaattgcatgaagaatctgcttagggttaggcgttttgcgctgcttcgcgatgtacgggccagatatacgcgttgacattgattattgactagttattaatagtaatcaattacggggtcattagttcatagcccatatatggagttccgcgttacataacttacggtaaatggcccgcctggctgaccgcccaacgacccccgcccattgacgtcaataatgacgtatgttcccatagtaacgccaatagggactttccattgacgtcaatgggtggagtatttacggtaaactgcccacttggcagtacatcaagtgtatcatatgccaagtacgccccctattgacgtcaatgacggtaaatggcccgcctggcattatgcccagtacatgaccttatgggactttcctacttggcagtacatctacgtattagtcatcgctattaccatggtgatgcggttttggcagtacatcaatgggcgtggatagcggtttgactcacggggatttccaagtctccaccccattgacgtcaatgggagtttgttttggcaccaaaatcaacgggactttccaaaatgtcgtaacaactccgccccattgacgcaaatgggcggtaggcgtgtacggtgggaggtctatataagcagagctctctggctaactagagaacccactgcttactggcttatcgaaattaatacgactcactatagggagacccaagctggctagcgtttaaacgggccctctagactcgagcggccgccactgtgctggatatctgcagaattccaccacactggactagtggatccgagctcggtaccaagcttaagtttaaaccgctgatcagcctcgactgtgccttctagttgccagccatctgttgtttgcccctcccccgtgccttccttgaccctggaaggtgccactcccactgtcctttcctaataaaatgaggaaattgcatcgcattgtctgagtaggtgtcattctattctggggggtggggtggggcaggacagcaagggggaggattgggaagacaatagcaggcatgctggggatgcggtgggctctatggcttctgaggcggaaagaaccagctggggctctagggggtatccccacgcgccctgtagcggcgcattaagcgcggcgggtgtggtggttacgcgcagcgtgaccgctacacttgccagcgccctagcgcccgctcctttcgctttcttcccttcctttctcgccacgttcgccggctttccccgtcaagctctaaatcgggggctccctttagggttccgatttagtgctttacggcacctcgaccccaaaaaacttgattagggtgatggttcacgtagtgggccatcgccctgatagacggtttttcgccctttgacgttggagtccacgttctttaatagtggactcttgttccaaactggaacaacactcaaccctatctcggtctattcttttgatttataagggattttgccgatttcggcctattggttaaaaaatgagctgatttaacaaaaatttaacgcgaattaattctgtggaatgtgtgtcagttagggtgtggaaagtccccaggctccccagcaggcagaagtatgcaaagcatgcatctcaattagtcagcaaccaggtgtggaaagtccccaggctccccagcaggcagaagtatgcaaagcatgcatctcaattagtcagcaaccatagtcccgcccctaactccgcccatcccgcccctaactccgcccagttccgcccattctccgccccatggctgactaattttttttatttatgcagaggccgaggccgcctctgcctctgagctattccagaagtagtgaggaggcttttttggaggcctaggcttttgcaaaaagctcccgggagcttgtatatccattttcggatctgatcaagagacaggatgaggatcgtttcgcatgattgaacaagatggattgcacgcaggttctccggccgcttgggtggagaggctattcggctatgactgggcacaacagacaatcggctgctctgatgccgccgtgttccggctgtcagcgcaggggcgcccggttctttttgtcaagaccgacctgtccggtgccctgaatgaactgcaggacgaggcagcgcggctatcgtggctggccacgacgggcgttccttgcgcagctgtgctcgacgttgtcactgaagcgggaagggactggctgctattgggcgaagtgccggggcaggatctcctgtcatctcaccttgctcctgccgagaaagtatccatcatggctgatgcaatgcggcggct  gcatacgcttgatccggctacctgcccattcgaccaccaagcgaaacatcgcatcgagcgagcacgtactcggatggaag  ccggtcttgtcgatcaggatgatctggacgaagagcatcaggggctcgcgccagccgaactgttcgccaggctcaaggcg  cgcatgcccgacggcgaggatctcgtcgtgacccatggcgatgcctgcttgccgaatatcatggtggaaaatggccgctttt  ctggattcatcgactgtggccggctgggtgtggcggaccgctatcaggacatagcgttggctacccgtgatattgctgaaga  gcttggcggcgaatgggctgaccgcttcctcgtgctttacggtatcgccgctcccgattcgcagcgcatcgccttctatcgcctt  cttgacgagttcttctgagcgggactctggggttcgaaatgaccgaccaagcgacgcccaacctgccatcacgagatttcg  attccaccgccgccttctatgaaaggttgggcttcggaatcgttttccgggacgccggctggatgatcctccagcgcggggat  ctcatgctggagttcttcgcccaccccaacttgtttattgcagcttataatggttacaaataaagcaatagcatcacaaatttcac  aaataaagcatttttttcactgcattctagttgtggtttgtccaaactcatcaatgtatcttatcatgtctgtataccgtcgacctctag  ctagagcttggcgtaatcatggtcatagctgtttcctgtgtgaaattgttatccgctcacaattccacacaacatacgagccgga  agcataaagtgtaaagcctggggtgcctaatgagtgagctaactcacattaattgcgttgcgctcactgcccgctttccagtcg  ggaaacctgtcgtgccagctgcattaatgaatcggccaacgcgcggggagaggcggtttgcgtattgggcgctcttccgctt  cctcgctcactgactcgctgcgctcggtcgttcggctgcggcgagcggtatcagctcactcaaaggcggtaatacggttatccacagaatcaggggataacgcaggaaagaacatgtgagcaaaaggccagcaaaaggccaggaaccgtaaaaaggcc  gcgttgctggcgtttttccataggctccgcccccctgacgagcatcacaaaaatcgacgctcaagtcagaggtggcgaaac  ccgacaggactataaagataccaggcgtttccccctggaagctccctcgtgcgctctcctgttccgaccctgccgcttaccgg  atacctgtccgcctttctcccttcgggaagcgtggcgctttctcatagctcacgctgtaggtatctcagttcggtgtaggtcgttcg  ctccaagctgggctgtgtgcacgaaccccccgttcagcccgaccgctgcgccttatccggtaactatcgtcttgagtccaacc  cggtaagacacgacttatcgccactggcagcagccactggtaacaggattagcagagcgaggtatgtaggcggtgctac  agagttcttgaagtggtggcctaactacggctacactagaagaacagtatttggtatctgcgctctgctgaagccagttacctt  cggaaaaagagttggtagctcttgatccggcaaacaaaccaccgctggtagcggtttttttgtttgcaagcagcagattacgc  gcagaaaaaaaggatctcaagaagatcctttgatcttttctacggggtctgacgctcagtggaacgaaaactcacgttaagg  gattttggtcatgagattatcaaaaaggatcttcacctagatccttttaaattaaaaatgaagttttaaatcaatctaaagtatatat  gagtaaacttggtctgacagttaccaatgcttaatcagtgaggcacctatctcagcgatctgtctatttcgttcatccatagttgcc  tgactccccgtcgtgtagataactacgatacgggagggcttaccatctggccccagtgctgcaatgataccgcgagaccca  cgctcaccggctccagatttatcagcaataaaccagccagccggaagggccgagcgcagaagtggtcctgcaactttatc  cgcctccatccagtctattaattgttgccgggaagctagagtaagtagttcgccagttaatagtttgcgcaacgttgttgccattg  ctacaggcatcgtggtgtcacgctcgtcgtttggtatggcttcattcagctccggttcccaacgatcaaggcgagttacatgatc  ccccatgttgtgcaaaaaagcggttagctccttcggtcctccgatcgttgtcagaagtaagttggccgcagtgttatcactcatg  gttatggcagcactgcataattctcttactgtcatgccatccgtaagatgcttttctgtgactggtgagtactcaaccaagtcattct  gagaatagtgtatgcggcgaccgagttgctcttgcccggcgtcaatacgggataataccgcgccacatagcagaactttaa  aagtgctcatcattggaaaacgttcttcggggcgaaaactctcaaggatcttaccgctgttgagatccagttcgatgtaaccca  ctcgtgcacccaactgatcttcagcatcttttactttcaccagcgtttctgggtgagcaaaaacaggaaggcaaaatgccgca  aaaaagggaataagggcgacacggaaatgttgaatactcatactcttcctttttcaatattattgaagcatttatcagggttattg  tctcatgagcggatacatatttgaatgtatttagaaaaataaacaaataggggttccgcgcacatttccccgaaaagtgccac  ctgacgtc  GV141-m*Rab5a*  gacggatcgggagatctcccgatcccctatggtgcactctcagtacaatctgctctgatgccgcatagttaagccagtatctgc  tccctgcttgtgtgttggaggtcgctgagtagtgcgcgagcaaaatttaagctacaacaaggcaaggcttgaccgacaattg  catgaagaatctgcttagggttaggcgttttgcgctgcttcgcgatgtacgggccagatatacgcgttgacattgattattgacta  gttattaatagtaatcaattacggggtcattagttcatagcccatatatggagttccgcgttacataacttacggtaaatggcccg  cctggctgaccgcccaacgacccccgcccattgacgtcaataatgacgtatgttcccatagtaacgccaatagggactttcc  attgacgtcaatgggtggagtatttacggtaaactgcccacttggcagtacatcaagtgtatcatatgccaagtacgcccccta  ttgacgtcaatgacggtaaatggcccgcctggcattatgcccagtacatgaccttatgggactttcctacttggcagtacatcta  cgtattagtcatcgctattaccatggtgatgcggttttggcagtacatcaatgggcgtggatagcggtttgactcacggggatttc  caagtctccaccccattgacgtcaatgggagtttgttttggcaccaaaatcaacgggactttccaaaatgtcgtaacaactcc  gccccattgacgcaaatgggcggtaggcgtgtacggtgggaggtctatataagcagagctctctggctaactagagaacc  cactgcttactggcttatcgaaattaatacgactcactatagggagacccaagctggctagcgtttaaacgggccctctagac  tcgagcggccgccactgtgctggatatctgcagaattcgccaccatggctaatcgaggagcaacaagacccaacgggcc  aaatactggaaataaaatatgccagttcaaactggtccttctaggagagtctgctgttggcaaatcaagcctggttcttcgcttt  gtgaaaggccaatttcatgaatttcaagagagtaccattggggctgcctttctaacccaaactgtgtgtcttgatgacacaaca  gtaaaatttgaaatatgggatacagctggtcaagaacggtatcatagcttagcaccaatgtactaccgaggagcacaagca  gccatagttgtgtatgatatcacaaatgaggaatcctttgcgagagcaaaaaactgggttaaagaacttcaaaggcaagca  agtcctaatattgtgatagctttgtcaggaaacaaagctgacttagcaaataaaagagctgttgacttccaggaagcacagtc  ctatgcagatgacaacagcttattatttatggagacatcagctaagacatcaatgaatgtaaatgaaatatttatggcaatagc  taaaaagctgccaaagaatgaaccacagaatcctggtgcaaactcagccagaggacgaggagtagaccttactgagcc  tgcacagccagccagaagccagtgttgtagtaacggaggtggaggatcagaacaaaaactcatctcagaagaggatctg  tgaggtaccaagcttaagtttaaaccgctgatcagcctcgactgtgccttctagttgccagccatctgttgtttgcccctcccccg  tgccttccttgaccctggaaggtgccactcccactgtcctttcctaataaaatgaggaaattgcatcgcattgtctgagtaggtgt  cattctattctggggggtggggtggggcaggacagcaagggggaggattgggaagacaatagcaggcatgctggggatg  cggtgggctctatggcttctgaggcggaaagaaccagctggggctctagggggtatccccacgcgccctgtagcggcgca  ttaagcgcggcgggtgtggtggttacgcgcagcgtgaccgctacacttgccagcgccctagcgcccgctcctttcgctttcttc  ccttcctttctcgccacgttcgccggctttccccgtcaagctctaaatcgggggctccctttagggttccgatttagtgctttacggc  acctcgaccccaaaaaacttgattagggtgatggttcacgtagtgggccatcgccctgatagacggtttttcgccctttgacgtt  ggagtccacgttctttaatagtggactcttgttccaaactggaacaacactcaaccctatctcggtctattcttttgatttataaggg  attttgccgatttcggcctattggttaaaaaatgagctgatttaacaaaaatttaacgcgaattaattctgtggaatgtgtgtcagtt  agggtgtggaaagtccccaggctccccagcaggcagaagtatgcaaagcatgcatctcaattagtcagcaaccaggtgtg  gaaagtccccaggctccccagcaggcagaagtatgcaaagcatgcatctcaattagtcagcaaccatagtcccgccccta  actccgcccatcccgcccctaactccgcccagttccgcccattctccgccccatggctgactaattttttttatttatgcagaggc  cgaggccgcctctgcctctgagctattccagaagtagtgaggaggcttttttggaggcctaggcttttgcaaaaagctcccgg  gagcttgtatatccattttcggatctgatcaagagacaggatgaggatcgtttcgcatgattgaacaagatggattgcacgca  ggttctccggccgcttgggtggagaggctattcggctatgactgggcacaacagacaatcggctgctctgatgccgccgtgtt  ccggctgtcagcgcaggggcgcccggttctttttgtcaagaccgacctgtccggtgccctgaatgaactgcaggacgaggc  agcgcggctatcgtggctggccacgacgggcgttccttgcgcagctgtgctcgacgttgtcactgaagcgggaagggactg  gctgctattgggcgaagtgccggggcaggatctcctgtcatctcaccttgctcctgccgagaaagtatccatcatggctgatg  caatgcggcggctgcatacgcttgatccggctacctgcccattcgaccaccaagcgaaacatcgcatcgagcgagcacgt  actcggatggaagccggtcttgtcgatcaggatgatctggacgaagagcatcaggggctcgcgccagccgaactgttcgc  caggctcaaggcgcgcatgcccgacggcgaggatctcgtcgtgacccatggcgatgcctgcttgccgaatatcatggtgg  aaaatggccgcttttctggattcatcgactgtggccggctgggtgtggcggaccgctatcaggacatagcgttggctacccgt  gatattgctgaagagcttggcggcgaatgggctgaccgcttcctcgtgctttacggtatcgccgctcccgattcgcagcgcatc  gccttctatcgccttcttgacgagttcttctgagcgggactctggggttcgaaatgaccgaccaagcgacgcccaacctgcca  tcacgagatttcgattccaccgccgccttctatgaaaggttgggcttcggaatcgttttccgggacgccggctggatgatcctcc  agcgcggggatctcatgctggagttcttcgcccaccccaacttgtttattgcagcttataatggttacaaataaagcaatagcat  cacaaatttcacaaataaagcatttttttcactgcattctagttgtggtttgtccaaactcatcaatgtatcttatcatgtctgtatacc  gtcgacctctagctagagcttggcgtaatcatggtcatagctgtttcctgtgtgaaattgttatccgctcacaattccacacaaca  tacgagccggaagcataaagtgtaaagcctggggtgcctaatgagtgagctaactcacattaattgcgttgcgctcactgcc  cgctttccagtcgggaaacctgtcgtgccagctgcattaatgaatcggccaacgcgcggggagaggcggtttgcgtattggg  cgctcttccgcttcctcgctcactgactcgctgcgctcggtcgttcggctgcggcgagcggtatcagctcactcaaaggcggta  atacggttatccacagaatcaggggataacgcaggaaagaacatgtgagcaaaaggccagcaaaaggccaggaacc  gtaaaaaggccgcgttgctggcgtttttccataggctccgcccccctgacgagcatcacaaaaatcgacgctcaagtcaga  ggtggcgaaacccgacaggactataaagataccaggcgtttccccctggaagctccctcgtgcgctctcctgttccgaccct  gccgcttaccggatacctgtccgcctttctcccttcgggaagcgtggcgctttctcatagctcacgctgtaggtatctcagttcgg  tgtaggtcgttcgctccaagctgggctgtgtgcacgaaccccccgttcagcccgaccgctgcgccttatccggtaactatcgtc  ttgagtccaacccggtaagacacgacttatcgccactggcagcagccactggtaacaggattagcagagcgaggtatgta  ggcggtgctacagagttcttgaagtggtggcctaactacggctacactagaagaacagtatttggtatctgcgctctgctgaa  gccagttaccttcggaaaaagagttggtagctcttgatccggcaaacaaaccaccgctggtagcggtttttttgtttgcaagca  gcagattacgcgcagaaaaaaaggatctcaagaagatcctttgatcttttctacggggtctgacgctcagtggaacgaaaa  ctcacgttaagggattttggtcatgagattatcaaaaaggatcttcacctagatccttttaaattaaaaatgaagttttaaatcaat  ctaaagtatatatgagtaaacttggtctgacagttaccaatgcttaatcagtgaggcacctatctcagcgatctgtctatttcgttc  atccatagttgcctgactccccgtcgtgtagataactacgatacgggagggcttaccatctggccccagtgctgcaatgatac  cgcgagacccacgctcaccggctccagatttatcagcaataaaccagccagccggaagggccgagcgcagaagtggtc  ctgcaactttatccgcctccatccagtctattaattgttgccgggaagctagagtaagtagttcgccagttaatagtttgcgcaac  gttgttgccattgctacaggcatcgtggtgtcacgctcgtcgtttggtatggcttcattcagctccggttcccaacgatcaaggcg  agttacatgatcccccatgttgtgcaaaaaagcggttagctccttcggtcctccgatcgttgtcagaagtaagttggccgcagt  gttatcactcatggttatggcagcactgcataattctcttactgtcatgccatccgtaagatgcttttctgtgactggtgagtactca  accaagtcattctgagaatagtgtatgcggcgaccgagttgctcttgcccggcgtcaatacgggataataccgcgccacata  gcagaactttaaaagtgctcatcattggaaaacgttcttcggggcgaaaactctcaaggatcttaccgctgttgagatccagtt  cgatgtaacccactcgtgcacccaactgatcttcagcatcttttactttcaccagcgtttctgggtgagcaaaaacaggaagg  caaaatgccgcaaaaaagggaataagggcgacacggaaatgttgaatactcatactcttcctttttcaatattattgaagcatt  tatcagggttattgtctcatgagcggatacatatttgaatgtatttagaaaaataaacaaataggggttccgcgcacatttcccc  gaaaagtgccacctgacgtc | |
